# Development and validation of a preoperative prediction model for colorectal cancer T-staging based on MDCT images and clinical information

**DOI:** 10.18632/oncotarget.19427

**Published:** 2017-07-21

**Authors:** Sha Sa, Jing Li, Xiaodong Li, Yongrui Li, Xiaoming Liu, Defeng Wang, Huimao Zhang, Yu Fu

**Affiliations:** ^1^ Department of Radiology, The First Hospital of Jilin University, Changchun, China; ^2^ College of Electronic Science and Engineering, Jilin University, Changchun, China; ^3^ Research Center for Medical Image Computing, Department of Imaging and Interventional Radiology, The Chinese University of Hong Kong, Shatin, New Territories, Hong Kong, China; ^4^ Union Medical Imaging Research Institute, Shenzhen, China

**Keywords:** colorectal cancer, T-staging, model, development and validation, random forest

## Abstract

**Objectives:**

This study aimed to establish and evaluate the efficacy of a prediction model for colorectal cancer T-staging.

**Results:**

T-staging was positively correlated with the level of carcinoembryonic antigen (CEA), expression of carbohydrate antigen 19-9 (CA19-9), wall deformity, blurred outer edges, fat infiltration, infiltration into the surrounding tissue, tumor size and wall thickness. Age, location, enhancement rate and enhancement homogeneity were negatively correlated with T-staging. The predictive results of the model were consistent with the pathological gold standard, and the kappa value was 0.805. The total accuracy of staging improved from 51.04% to 86.98% with the proposed model.

**Materials and Methods:**

The clinical, imaging and pathological data of 611 patients with colorectal cancer (419 patients in the training group and 192 patients in the validation group) were collected. A spearman correlation analysis was used to validate the relationship among these factors and pathological T-staging. A prediction model was trained with the random forest algorithm. T staging of the patients in the validation group was predicted by both prediction model and traditional method. The consistency, accuracy, sensitivity, specificity and area under the curve (AUC) were used to compare the efficacy of the two methods.

**Conclusions:**

The newly established comprehensive model can improve the predictive efficiency of preoperative colorectal cancer T-staging.

## INTRODUCTION

Colorectal cancer is the third most common cancer worldwide, and the incidence and mortality rates of colorectal cancer continue to increase in China. Approximately 376.3 thousand new cases of colorectal cancer were diagnosed in 2015, and 191 thousand patients died from this cancer in China [1]. Therefore, the early detection of colon cancer and the selection of optimal treatment are particularly important, especially the prediction of tumor stage because it determines the administration of adjuvant therapy. At present, the treatment of colorectal cancer is mainly radical surgery and adjuvant therapy. The development of treatment strategies rely on patients’ TNM staging. Accurate preoperative staging can provide an objective basis for the adjuvant treatment of colorectal cancer, the choice of surgical protocols and prognosis [2]. Therefore, preoperative staging of colorectal cancer is particularly important.

Recent advances and continued progress in cross-sectional radiologic imaging of the colorectum have improved the noninvasive evaluation of colorectal tumors [3]. Moreover, computed tomographic colonography (CTC) permits additional image reconstruction techniques. Currently, CTC is the most common radiological examination method for suspected colorectal disease because of its high sensitivity for colorectal cancer [4, 5]. Specifically, CTC provides imaging information for the primary tumor, local lymph node metastasis and distant metastasis [6]. In the clinical routine radiologists usually solely use the AJCC tumor TNM system to predict the preoperative staging of colorectal cancer base on CT images. However, the traditional method for colorectal tumor T-staging used by radiologists maybe overestimated or underestimated due to individual subjectivity in the assessment.

The random forest (RF) algorithm is based on a popular statistical learning theory that uses the bootstrap resampling method to extract multiple samples from the original sample. Compared with traditional classification methods, such as linear regression analysis and logistic regression analysis, RF has no restrictions on the number of predictor variables, no collinearity, and deals with complex nonlinear relationships. Therefore, this study aimed to develop a comprehensive model by random forest that accurately predicts the T-staging of colorectal cancer.

## RESULTS

### Clinical characteristics

This study included 611 patients with 611 colorectal cancer lesions confirmed by pathology. All patients underwent conventional CTC or water enema MDCT prior to surgery. The characteristics of 611 colorectal cancer patients are given in Table 1. The average age of the patients was 60.8 ± 10.8 (28–93) years old, and the male to female ratio was 1.24:1. Tumors were more common in the left colon (76.92%), and the predilection sites were the rectum (38.8%) and sigmoid colon (29.0%). Tumor staging was stratified as follows: ≤ T2 stage in 212 (34.7%) patients, T3 stage in 221 (36.17%) patients, and T4 stage in 178 (29.13%) patients. Thirteen factors from the clinical information and preoperative CTC images and one dependent variable from the pathological results were counted in this study. Except gender, T-staging correlated with twelve independent variables. T-staging positively correlated with the CEA level (*ρ =* 0.423, *p <* 0.001), expression of CA19-9 (*ρ =* 0.305, *p <* 0.001), wall deformity (*ρ =* 0.642, *p <* 0.001), blurred outer edge of the intestine (*ρ =* 0.486, *p <* 0.001), fat infiltration (*ρ =* 0.597, *p <* 0.001), infiltration into the surrounding tissue (*ρ =* 0.296, *p <* 0.001), tumor size (*ρ =* 0.547, *p <* 0.001), and wall thickness (*ρ =* 0.335, *p <* 0.001). Conversely, age (*ρ =* −0.111, *p* = 0.006), tumor location (*ρ =* −0.28, *p <* 0.001), enhancement rate (*ρ =* −0.103, *p* = 0.011) and enhancement homogeneity (*ρ =* −0.354, *p <* 0.001) were negatively correlated with T-staging.

**Table 1:** Characteristics of all included patients

Independent variables		≤ T2	T3	T4	p^a^
Gender, No. (%)	Male	114(53.8)	130(58.8)	94(52.8)	0.929
	Female	98(46.2)	91(41.2)	84(47.2)	
Age, year, mean ± SD		62.2 ± 10	60.6 ± 10.7	59.2±11.7	0.006*
CEA, ng/ml, M(P_25_-P_75_)		2.18(1.17–3.34)	3.82(2.14–9.22)	6.26(2.79–21.17)	< 0.001*
CA19–9, ng/ml, M(P_25_-P_75_)		9.6(5.88–14.25)	12.47(7.03–23.26)	19.63(9.01–81.44)	< 0.001*
Location, No. (%)	Right	23(10.8)	45(20.4)	73(41)	< 0.001*
	Left	189(89.2)	176(79.6)	105(59)	
Deformity, No. (%)	1	18(8.5)	1(0.4)	0	< 0.001*
	2	74(34.9)	25(11.3)	0	
	3	82(38.7)	28(12.7)	9(5.1)	
	4	38(17.9)	167(75.6)	169(94.9)	
Blurred outer edge, No. (%)	Absent	123(58)	35(15.8)	9(5.1)	< 0.001*
	Present	89(42)	186(84.2)	169(94.9)	
Fat infiltration, No. (%)	Absent	189(89.2)	81(36.7)	28(15.7)	< 0.001*
	Present	23(10.8)	140(63.3)	150(84.3)	
Infiltration into the surrounding tissue, No. (%)	Absent	212(100)	208(94.1)	141(79.2)	< 0.001*
	Present	0	13(5.9)	37(20.8)	
Size, cm, M(P_25_-P_75_)		2.6(1.8–3.8)	4.3(3.5–5.7)	5.3(4.1–7)	< 0.001*
Wall thickness, cm, M(P_25_-P_75_)		0.8(0.6–1.2)	1(0.8–1.2)	1.3(1–1.6)	< 0.001*
Enhancement rate, %, M(P_25_-P_75_)		0.89(0.66–1.14)	0.9(0.69–1.17)	0.82(0.55–1.01)	0.011*
Enhancement homogeneity, No. (%)	inhomogeneous	21(9.9)	47(21.3)	88(49.4)	< 0.001*
	homogeneous	191(90.1)	174(78.7)	90(50.6)	

NOTE. Abbreviations: CEA, carcinoembryonic antigen. CA19-9, carbohydrate antigen 19-9.

Enumeration data is expressed as a percentage. Normal distribution of measurement data is expressed as mean ± SD. Non-normal distribution of measurement data is expressed as M(P25-P75).

*p* value is derived from univariate association analyses between each of the independent variables and pathological T-staging.

^a^Spearman correlation analysis. * *p* value < 0.05.

The cohort is divided into the training group (419 patients) and validation group (192 patients), and the basic clinical information of the two groups is provided below (Table 2). The training group and validation group did not differ in gender, age or T-stage (*p* = 0.23–0.573). The model can be validated using this validation group's data set.

**Table 2:** Clinicopathologic information of patients in the training and validation groups

		Training group	Validation group	*p*
Gender, No. (%)	male	235 (56.1)	103 (53.6)	0.573^a^
	female	184 (43.9)	89 (46.4)	
Age, year, mean ± SD		61.1 ± 10.9	60 ± 10.7	0.23^b^
T-stage	≤ T2	149 (35.6)	63 (32.8)	0.399^a^
	T3	155 (37)	66 (34.4)	
	T4	115 (27.4)	63 (32.8)	

NOTE. *p* value < 0.05 illustrates a significant difference between two groups.

a chi-square test. b *t*-test.

### Validation of the prediction model performance

**Figure 1:**
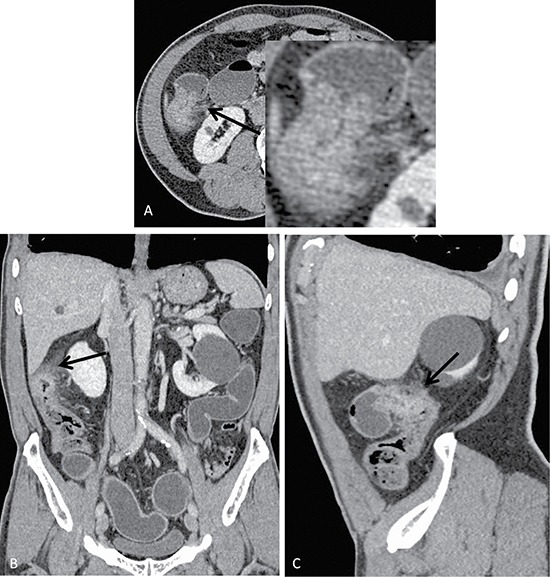
Axial (**A**), coronal (**B**) and sagittal (**C**) images of a colon cancer lesion located in the hepatic flexure of the colon of a 72-year-old male who presented with a change in defecation habits. Conventional methods preoperatively predicted the T-stage as T4 because of evident serosa thickening, increased surrounding fat gap density, a blurred neighboring peritoneal border, and adjacent peritoneal thickening on the CT image. Conversely, the model predicted T3 disease, which was consistent with the pathological staging.

The results of the model-predicted colorectal cancer T-staging were highly consistent with the results of the pathological gold standard and much more accurate than traditional methods (Table 3). The model correctly predicted the T-stage of the validation cohort for 167 of 192 (86.98%) patients, whereas the traditional method correctly predicted the T-stage for only 98 patients (51.04%), this difference was significant (χ^2^ = 57.974, *p <* 0.001). Moreover, the prediction of the model for ≤ T2 and T3 disease was more accurate than that of the traditional method (χ^2^ = 24.738, 39.6; *p <* 0.001, < 0.001), there is no statistical differences for T4 disease (χ^2^ = 3.316, *p* = 0.069). The model incorrectly overestimated and underestimated the stage for 17 of 25 patients (68%) and 8 patients (32%), respectively. Specifically, 10 patients with ≤ T2 disease were predicted to have stage T3 disease, and 7 patients with stage T3 disease were predicted to have T4 disease. Conversely, 5 patients with stage T3 disease were predicted to have ≤ T2 disease, and 3 patients with T4 disease were predicted to have T3 disease. Using the traditional method, the staging was incorrectly overestimated and underestimated for 81 of 94 patients (86.17%) and 13 patients (13.83%), respectively. Figure 1 shows a pathologically confirmed T3 colorectal cancer lesion that was misdiagnosed as a T4 lesion by traditional method because the tumor exhibited a strip-like, high-density appearance in the peripheral adipose tissue. The model classified this lesion as T3 stage correctly. Thus the opacity of the tumor surrounding fat is not a tumor infiltration, but an inflammatory response.

**Table 3:** Model's and traditional method's prediction results

**Model, No. (%)**	**Pathological staging**					
	**≤ T2**	**T3**	**T4**	**Total**	**consistency**	
					**Kappa**	***p***
≤ T2	53 (84.1)	5 (7.6)	0	58 (30.2)	0.819^a^	< 0.001*
T3	10 (15.9)	54 (81.8)	3 (4.8)	67 (34.9)	0.712	< 0.001*
T4	0	7 (10.6)	60 (95.2)	67 (34.9)	0.884^a^	< 0.001*
Total	63 (100)	66 (100)	63 (100)	192 (100)	0.805^a^	< 0.001*
**Traditional method, No. (%)**	**Pathological staging**					
	**≤ T2**	**T3**	**T4**	**Total**	**consistency**	
					**Kappa**	***p***
≤ T2	26 (41.3)	4 (6.1)	1 (1.6)	31 (16.1)	0.434	< 0.001*
T3	31 (49.2)	18 (27.3)	8 (12.7)	57 (29.7)	–0.038	0.596
T4	6 (9.5)	44 (66.7)	54 (69.6)	104 (54.2)	0.403	< 0.001*
Total	63 (100)	66 (100)	63 (100)	192 (100)	0.266	< 0.001*

NOTE. *p value < 0.05. ^a^Kappa value > 0.8.

The sensitivity and specificity of colorectal cancer staging predicted by the model and conventional methods are shown in the table below (Table 4). The sensitivity, specificity and accuracy of the model are higher than traditional method which has certain significance in accurate preoperative classification and treatment options for colorectal cancer.

**Table 4:** Sensitivity and specificity of the model and traditional method for predicting the T-stage

model			traditional method	
	Sensitivity (%)	Specificity (%)	Sensitivity (%)	Specificity (%)
≤ T2	84.1	96.1	41.3	96.3
T3	81.8	89.7	27.3	69
T4	95.2	94.6	85.7	61.2

**Figure 2:**
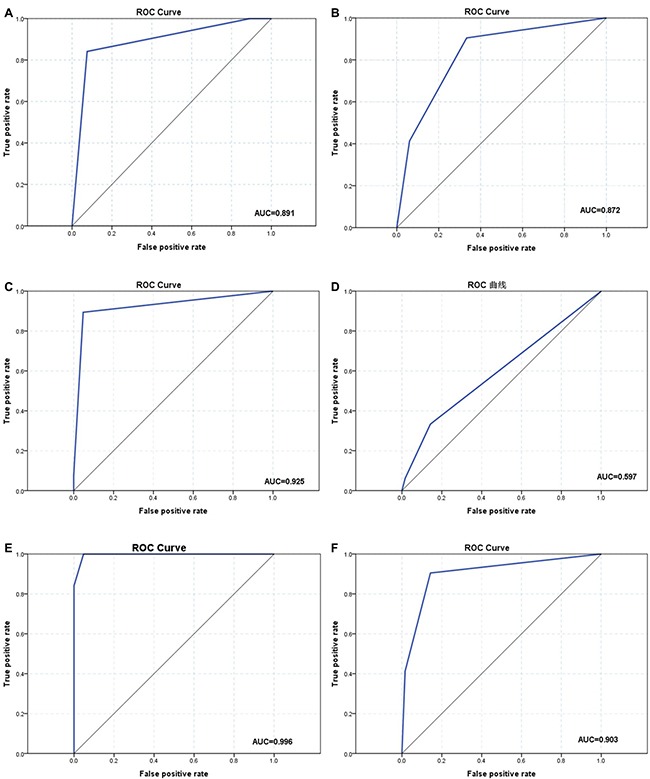
Receiver operating characteristic (ROC) curves and area under the curve (AUC) of the model and conventional method by stage (**A**, **C**, **E**) The ROC curves of model (a: ≤ T2 and T3 stage; c: T3 and T4 stage; e: T2 and T4 stage). (**B**, **D**, **F**) The ROC curves of the conventional method (b: ≤ T2 and T3 stage; d: T3 and T4 stage; f: T2 and T4 stage).

The ROC curves and AUC values of the two prediction methods are shown below in Figure 2. The AUC for the accuracy of prediction result was significantly higher in the model than in the traditional method.

## DISCUSSION

Colorectal cancer has a high incidence and mortality [7]. With the continuous development of colorectal cancer treatment methods, many strategies are available for the treatment of colorectal cancer, including local excision, radical resection, and multimodal therapy [8, 9]. The rapid development of surgical techniques has placed surgery as the leading treatment for colorectal cancer. But a specific treatment plan strongly depends on accurate pretreatment staging [2, 10, 11]. In surgical treatment, stage T1 tumors are recommended for local excision and radical resection for stage T2-4 tumors. Surgical procedures include open and laparoscopic colorectal cancer surgery. Laparoscopic surgery has been shown to improve short-term clinical and oncologic outcomes [12, 13], including shortened hospital stays, fewer postoperative complications and accelerated rehabilitation [14]. Thus, laparoscopic-assisted colectomy has been widely accepted for the treatment of mucosal or submucosal carcinoma, whereas open surgery is preferably used to treat advanced cancer [15, 16]. In the medical treatment, it is recommended stage T3 and T4 tumor patients with neoadjuvant therapy. Neoadjuvant radiotherapy and chemotherapy can shrink T4 stage colorectal tumors to allow sphincter-preserving surgery, which reduces the recurrence rate and improves the survival rate [17]. But, adjuvant therapy is not recommended for patients with stage ≤T2 without lymph node metastasis and distant metastasis. In addition, tumor T-staging is the strongest prognostic factor for colorectal cancer survivors [18]. Therefore, precise staging of colorectal cancer is used to determine the most appropriate treatment strategy and evaluate the outcome of therapy.

Several diagnostic tools are available for the diagnosis and evaluation of colorectal cancer. Colonoscopy and biopsy are the gold standard for the diagnosis of colorectal cancer. However, it also has some limitations. For the incomplete colonoscopy due to distal obstruction, the proximal colorectal segment cannot be effectively detected. It is also not able to detect extra intestinal conditions and is deficient in the evaluation of colorectal cancer TNM staging. In non-invasive imaging methods, magnetic resonance (MR) techniques feature a higher soft tissue resolution and can clearly distinguish rectal intestinal wall stratification [19]. However the diagnostic efficacy for the colon is low because this technique is time-consuming and cost-ineffective. Thus, multi-slice spiral CT remains one of the best methods for the preoperative staging of colorectal cancer [20, 21]. Advances in hardware and post-processing software technology have improved the accuracy of colorectal cancer screening, staging and monitoring and provide a wealth of information for the preoperative assessment of colorectal cancer T-staging [19, 20, 22]. Therefore, the use of MDCT for accurate preoperative staging can effectively help physicians to select appropriate therapeutic regimen, which has important reference value to clinical practice.

A number of studies have correlated wall deformations in the CT image with T-staging, and the accuracy of this approach ranges from 73%–83% [19]. However, a uniform criterion for determining tumor T-staging is lacking. Radiologists identify the T-stage of colorectal cancer using a traditional method based on the 7th edition of the AJCC tumor TNM system[23]. In clinical practice, the diagnosis of stage depends on the doctors’ experience in medical image knowledge and disease diagnosis, the results lack of quantitative analysis and objectivity. Thus, due to limitations in CT resolution and doctors’ subjectivity, radiologists cannot accurately identify tumors that surround connective tissue hyperplasia, inflammation and peritumoral fat infiltration [24]. Because of these shortcomings, the staging of many tumors was always incorrectly classified. In this study, the accuracy rate of tumor staging was only 51%. In particular, the accuracy rate of stage T2 and T3 is very low, only 39% and 27%. Therefore, it is very important to establish a new stage prediction model.

In recent years, the development of medical imaging technology has increased the amount of information provided by these images. However, general medical statistical analyses are not satisfactory, and the demand for data mining is growing. The RF method is based on a popular statistical learning theory that uses the bootstrap resampling method to extract multiple samples from the original sample. The forecast of multiple decision trees is then combined, and the final prediction result is obtained by voting. RF predictions are highly accurate and tolerant to exception values and noise without being prone to over-fitting. Thus, RF has been widely applied in medicine, bioinformatics, management and other fields [25, 26]. RF can be used for multivariable classification and prediction. Its biggest feature is suitable for analyzing data with complex nonlinear relationships. In this study we used the random forest method to establish a model to preoperatively predict the T-staging of colorectal cancer. The external validation method is used to evaluate the classification effect of the prediction model. The results showed that the overall accuracy of the prediction was 87%, whereas the accuracy for the prediction of each stage was 84%, 82%, and 95%, which was higher than the 73%–83% accuracy values reported in previous studies. The accuracy of the traditional method was lower than that reported in previous studies, whereas our model significantly improved the accuracy of the prediction for tumor T-staging. The model developed in this study could successfully and accurately predict preoperative colorectal cancer T-staging.

Nevertheless, this study was subject to the following limitations. Firstly, not all patients included in the study underwent CTC examination; some patients underwent MDCT water enema examination. In our analysis, we classified these two methods as one method. Although the research objective was independent of the two CT examination methods, the reconstruction technologies of these methods differ, which may have resulted in differences in the final experimental results. Specifically, CTC examination relies on ray sum imaging, which more accurately measures the tumor angle. Secondly, this study only considered T-staging and established a T-staging model. We did not examine N and M staging, which may have biased the discussion of tumor treatment. We hope to improve the TNM staging system in future studies to establish a complete tumor stage prediction model.

## MATERIALS AND METHODS

### Patients

This retrospective study was approved by the ethics committee of our institution and did not require informed consent. From January 2016 to April 2017, a total of 2482 patients underwent CTC and multidetector CT (MDCT) with water enema at our hospital. Of these patients, 611 patients who were suspected to have colorectal cancer (338 men, 273 women; mean age, 60.8 *±* 10.8 years; range, 28–93 years) accepted the surgical procedures and provided postoperative pathological results. All patients were randomly divided into two groups at a ratio of approximately 2:1. Specifically, 419 patients (235 men, 184 women; mean age, 61.1 *±* 11.9 years; range, 28–93 years) were enrolled in the training group, and 192 patients (103 men, 89 women; mean age, 60 *±* 10.7 years; range, 28–91 years) were enrolled in the validation group.

### CT scanning

All patients ate a semi-liquid dinner containing 750 ml of polyethylene glycol electrolyte powder (Shutaiqing, Staidson biological pharmaceutical Limited by Share Ltd, Beijing, China) in the evening on the day before the examination. The patients received another 1,500 ml of polyethylene glycol in the morning on the day of the examination. Smooth muscle relaxation was achieved with an intramuscular injection 15 minutes before the examination. Thereafter, room air was infused through a latex tube placed in the rectum with a manual insufflation device to distend the colon for patients who underwent CTC; patients who underwent MDCT received a water enema to expand the bowel.

Patients undergoing CTC were subjected to a double position scan. In the prone position, the patients underwent a low-dose scanning protocol (120 kVp, 25 mAs), whereas in the supine position plain and enhanced scan were performed by routine dose (120 kVp, 150 mAs). The patients undergoing MDCT after a water enema underwent a routine CT scanning protocol (120 kVp, 150 mAs) in the supine position with contrast-enhanced scanning. The following scan parameters were employed: pitch, 0.8; gantry rotation time, 0.5 s; slice thickness, 3.0 mm; tube voltage, 120 kVp; tube current, 25 mAs (prone) and 150 mAs (supine); and matrix, 512 × 512. CT images acquired in both the supine and prone positions were reconstructed at an interval of 1 mm, using iDose4 level-6 for the prone position and filtered back projection (FBP) for the supine position. Technologists reconstructed the CTC images using a Raysum reconstruction technique at a workstation (Ziostation; Ziosoft2, Tokyo, Japan). All images were transferred to a PC-based communication system (PACS).

### Data acquisition

A total of 13 predictive variables (clinical and imaging data) and one dependent variable (pathological results) needed to be collected from the model and validation cohorts of patients. The clinicopathologic data of patients, including gender, age, carcinoembryonic antigen (CEA) level, expression of carbohydrate antigen 19-9 (CA19-9), and pathological results were collected from case database. We reviewed the following nine imaging data from CT images: tumor location (left colon, right colon), tumor size, intestinal wall thickness of the lesion, wall deformity, contrast enhancement rate of the lesion, enhancement homogeneity, blurred outer edge of the intestine, pericolonic fat infiltration, and infiltration into the surrounding tissue.

**Figure 3:**
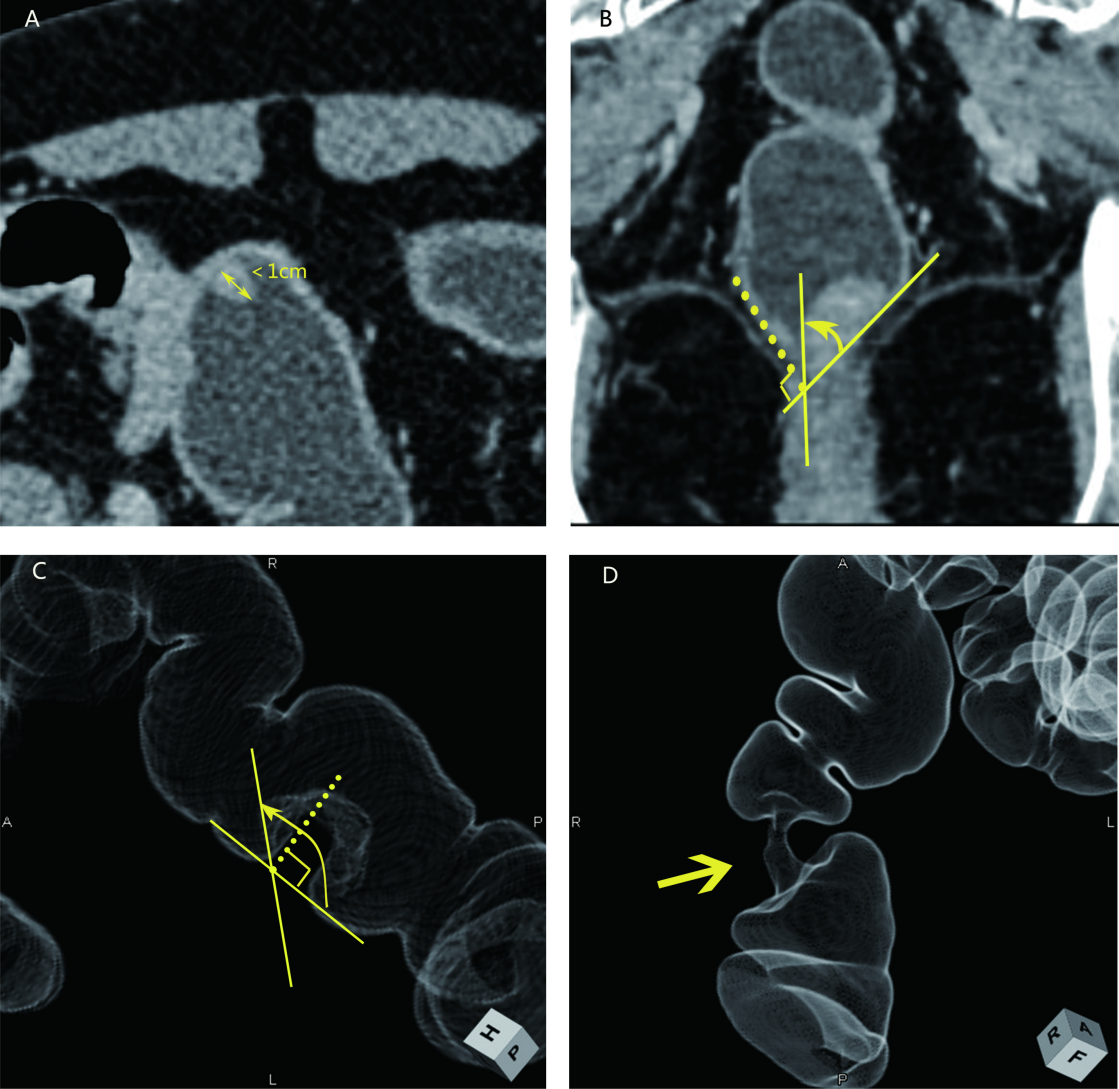
Four types of wall deformations revealed using MDCT following a water enema (**A**, **B**) and ray sum images (**C**, **D**). A: a score of 1 indicates a lesion smaller than 1 cm; B: a score of 2 indicates a lesion angle less than 90°; C: a score of 3 indicates a lesion angle greater than 90°; D: a score of 4 indicates the apple core sign intestinal wall deformation.

According to the classification method previously reported by Kazuhito Sato et al. [27], the wall deformity was scored from 1–4 as follows: 1 – lesions smaller than 1 cm and 2–4 – lesions with lengths equal to or greater than 1 cm. Specifically, a score of 2 represented an angle less than 90° formed by the outline of the lesion and the outer edge of the intestinal tract, whereas a score of 3 indicated an angle that was equal to or greater than 90°. The apple core sign was classified as a score of 4 (Figure 3). The ray sum images were used to show the best angle for measurement. In conventional CT images, we identified the largest dimension of the tumor by reconstruction of the original images and then measured the desired angle.

The contrast enhancement rate (CER) was calculated using the following formula (1). We selected the maximum dimension of the lesion from the plain phase and portal venous phase images to draw the region of interest (ROI) while avoiding cystic and necrotic zones and the transition zone between lesions and the normal intestinal canal. We measured the CT value of the ROI three times and calculated the average of the three results as follows.

$$\text{CER }=\;\frac{\text{CTN lesion portal - CTN lesion plain}}{\text{CTN lesion plain}}\times 100\%$$ (1)

Abbreviations: CER, contrast enhancement rate. CTN, CT number.

Based on a comprehensive review of the literature and tumor's evaluation by the 7th edition of the AJCC tumor TNM system, three radiological signs were reviewed: blurred outer edge of the intestine, pericolonic fat infiltration, and infiltration into the surrounding tissue. The above radiological signs were evaluated for given segments as follows: The presence and absence of any sign in a given segment was rated as ‘1’ and ‘0’, respectively. Similarly, enhancement homogeneity of a lesion was rated as ‘1’ and ‘0’, respectively. These clinicopathologic and imaging parameters are summarized in Supplementary Table 1. Table 5 shows the coding instructions for the predictor variables.

**Table 5:** Predictive variables, dependent variable and coding descriptions of model

number	variable	variable descriptions	number	variable	variable descriptions
1	Gender	0 = man; 1 = woman	1	Fat infiltration	0 = absence; 1 = presence
2	Age	continuous	2	Infiltration into the surrounding tissue	0 = absence; 1 = presence
3	CEA, ng/ml	continuous	3	Size, cm	continuous
4	CA19-9, ng/ml	continuous	4	Wall thickness, cm	continuous
5	Location	0 = right; 1 = left	5	Enhancement rate, %	continuous
6	Deformity	1 = ≤ 1 cm; 2 = < 90°; 3 = > 90°; 4 = apple core	6	Enhancement homogeneity	0= inhomogeneous; 1= homogeneous
7	Blurred outer edge	0 = absence; 1 = presence	7	T stage*	1 =≤ T2; 2 = T3; 3 = T4

*Dependent variable; The rest were predictor variables.

### Development and validation of a predictive model

In this study, random forest algorithm was used to establish the preoperative staging model of colorectal cancer. Traditional statistical classification methods such as linear regression analysis and logistic regression analysis have great limitation in the classification research. The traditional classification model is often not accurate enough and is prone to overfitting problems. Thus, many scholars raised the prediction accuracy by aggregating multiple models, which is called ensemble or classifier combination. Random forest is a machine learning and an integrated algorithm including multiple decision trees, the output of which is determined by the decision tree model [28, 29]. This method combines Breiman's thought of “Bootstrap Aggregating” and “Random Subspace Method” introduced by Ho [30].

The calculation process of the random forest classification model referred to literature [28, 31]. The basic idea of establishing random forest model is to continue to generate the training samples and test samples through the bootstrap resampling technique, and a number of classification trees are generated from the training samples to form a random forest. Then, the final classification results are obtained by combining the voting results of sub classifiers. The important parameter of random forest is the number of trees — ntree, in this study ntree = 100. The calculation process is as follows:

(1) The original training set is N (N = 419). N_x_ samples are randomly taken as a bootstrap sample, the size of which is the same as the input (N_x_ = N). Out-of-bag (OOB) consists of untaken samples. This is repeated S times to create S classification trees.

(2) There are m_a_ variables, m_try_ variables are randomly selected at each node of each tree (m_try_ < m_a_), in accordance with the principle of minimum impurity of the node to filter out the best splitting points for branch growth. In the entire forest establishing process m_try_ remains constant.

(3) RF is composed of the classification trees. The test data are discriminated and classified by RF classifier. The classification result depends on the number of voting by tree classifiers.

### Prediction of colorectal cancer staging using conventional method

**Figure 4:**
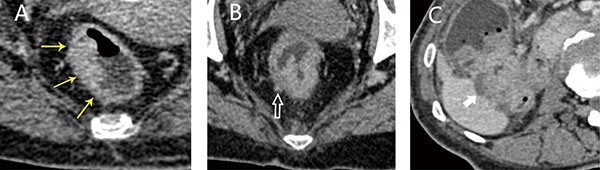
Axial images of tumors of various stages obtained using a traditional method (**A**) T2 stage, lesions exhibited a smooth outer edge; (**B**) T3 stage, the edge of the intestinal wall shows sharp corners; (**C**) T4 stage, the lesion had invaded the inferior margin of the adjacent liver.

The traditional colorectal cancer imaging staging method evaluates the deformation of the bowel wall at the lesion based on the AJCC tumor TNM system. Because of the low resolution of CT [32], T1 and T2 tumors were collectively referred to as ≤ T2 stage. According to previous studies [6, 18], CT images containing ≤ T2 tumors showed lesions with smooth outer edges of the intestinal wall, whereas T3 tumors exhibited rough serous layers with the presence of speculation. T4 tumors had infiltrated the peritumoral fat plane, as evidenced by streaks and nodules, or had infiltrated adjacent organs (Figure 4). According to this conventional method, three experienced radiologists staged the tumors of patients in the validation cohort independently. If their diagnosis was inconsistent, we adopted the result of their agreement or the conclusion of the radiologist with more experience.

### Statistical analysis

Univariate analyses were used to analyze the correlation between the dependent variable (pathological T-staging) and the independent variables (gender, age, CEA, CA19-9, tumor location, tumor size, intestinal wall thickness of lesion, wall deformity, contrast enhancement rate of the lesion, enhancement homogeneity, and tumor invasion sign). All data were analyzed using Spearman correlation analysis. A Chi-squared test and group *t*-test were used to analyze differences between the training and validation groups.

This study used the package random forest (RF) in MATLAB (2016a, MathWorks, USA) to train the T-stage classification model of colorectal cancer. The accuracy of the model and traditional method for predicting T-staging was evaluated based on a percentage, and the differences in these percentages were compared with the Chi-squared test. Pathological results were consistent with the models and the traditional methods. k < 0.4 is poor consistency, 0.4 ≤ k < 0.8 is medium consistency, k ≥ 0.8 is good consistency. The sensitivity and specificity of two method's predictive results were calculated. The area under the curve (AUC) of the receiver operating characteristic (ROC) curve for the proposed model and traditional method was calculated in terms of the preoperative prediction of colorectal cancer T-staging.

All data were analyzed with SPSS 18.0.0 software. Significance was set at *p <* 0.05.

## SUPPLEMENTARY MATERIALS TABLE




