# Supplementary material for: Development and validation of a preoperative prediction model for colorectal cancer T-staging based on MDCT images and clinical information
Source: Oncotarget. 2017 Jul 21;8(33):55308–18. doi: 10.18632/oncotarget.19427 (PMC5589660; doi:10.18632/oncotarget.19427)
Supplement: Supplementary file 1 [file oncotarget-08-55308-s001.pdf]

Development and validation of a preoperative prediction model for colorectal cancer T-staging based on MDCT images and clinical information

SUPPLEMENTARY MATERIALS

Supplementary Table 1: The clinical, imaging and pathological data of all the patients. See Supplementary_Table_1
