# Supplementary material for: Development and validation of a preoperative prediction model for colorectal cancer T-staging based on MDCT images and clinical information
Source: Oncotarget. 2017 Jul 21;8(33):55308–18. doi: 10.18632/oncotarget.19427 (PMC5589660; doi:10.18632/oncotarget.19427)
Supplement: Supplementary file 2 [file oncotarget-08-55308-s002.docx]

**Supplementary Table 1:The clinical, imaging and pathological data of all the patients**

| number | Gender | Age, year | CEA, ng/ml | CA19-9, ng/ml | Location | Deformity | Blurred outer edge | Fat infiltration | Infiltration into the surrounding tissue | Size, cm | Wall thickness, cm | Enhancement homogeneity | Enhancement rate, % | T-staging |
| --- | --- | --- | --- | --- | --- | --- | --- | --- | --- | --- | --- | --- | --- | --- |
| 1 | male | 74 | 3.8 | 10.23 | rectum | 4 | Present | Present | Absent | 5.2 | 1.3 | homogeneous | 0.75 | T3 |
| 2 | female | 70 | 19.69 | 0.6 | rectum | 4 | Present | Present | Absent | 5 | 0.5 | homogeneous | 1.71 | T3 |
| 3 | female | 60 | 1.87 | 9.63 | sigmoid | 3 | Present | Present | Absent | 4.4 | 2 | homogeneous | 1.13 | T3 |
| 4 | male | 66 | 8.65 | 8.65 | rectum | 2 | Absent | Absent | Absent | 2 | 1.2 | homogeneous | 0.4 | ≤T2 |
| 5 | female | 74 | 3.19 | 65.77 | sigmoid | 4 | Present | Present | Present | 6 | 1.6 | inhomogeneous | 0.88 | T3 |
| 6 | female | 63 | 8.47 | 24.94 | sigmoid | 4 | Present | Present | Present | 5 | 1.4 | homogeneous | 1.62 | T3 |
| 7 | male | 67 | 2.69 | 0.6 | rectum | 3 | Present | Present | Absent | 5 | 1 | homogeneous | 0.51 | T3 |
| 8 | male | 71 | 1.76 | 6.42 | sigmoid | 4 | Present | Absent | Absent | 3 | 0.6 | homogeneous | 0.4 | ≤T2 |
| 9 | male | 67 | 4.76 | 37.96 | sigmoid | 4 | Present | Present | Absent | 6 | 1.4 | inhomogeneous | 0.84 | T4 |
| 10 | male | 46 | 4.58 | 24.57 | rectum | 2 | Present | Present | Absent | 3.7 | 1.2 | homogeneous | 1.21 | T3 |
| 11 | male | 61 | 17.05 | 68.12 | sigmoid | 4 | Present | Present | Absent | 4 | 0.8 | inhomogeneous | 0.77 | T3 |
| 12 | male | 73 | 1.98 | 20.91 | cecum | 3 | Present | Absent | Absent | 2.8 | 0.6 | homogeneous | 0.77 | T3 |
| 13 | female | 58 | 4.12 | 118.1 | ascending | 4 | Present | Present | Absent | 7 | 1.6 | inhomogeneous | 0.48 | T4 |
| 14 | female | 70 | 3.13 | 5.96 | ascending | 4 | Present | Present | Absent | 5 | 1.2 | homogeneous | 0.94 | T4 |
| 15 | female | 63 | 2.11 | 15.92 | sigmoid | 4 | Present | Present | Absent | 4 | 0.6 | homogeneous | 0.8 | T3 |
| 16 | female | 64 | 1.81 | 19.51 | rectum | 3 | Present | Present | Absent | 4 | 0.6 | homogeneous | 0.69 | T3 |
| 17 | female | 66 | 4.98 | 8.86 | rectum | 3 | Present | Present | Absent | 3 | 1.2 | homogeneous | 0.5 | ≤T2 |
| 18 | male | 80 | 12.3 | 8.1 | sigmoid | 2 | Present | Present | Absent | 5 | 0.7 | inhomogeneous | 2.46 | T3 |
| 19 | female | 61 | 1.39 | 9.63 | cecum | 2 | Absent | Absent | Absent | 1.4 | 0.7 | homogeneous | 2.21 | ≤T2 |
| 20 | male | 66 | 1.27 | 10.54 | sigmoid | 4 | Present | Present | Absent | 10 | 2 | inhomogeneous | 0.15 | T4 |
| 21 | female | 61 | 233.21 | 29.17 | rectum | 4 | Present | Present | Present | 6 | 0.9 | inhomogeneous | 0.66 | T3 |
| 22 | male | 58 | 3.23 | 17.79 | sigmoid | 4 | Present | Present | Absent | 5 | 0.6 | homogeneous | 1.32 | T3 |
| 23 | male | 65 | 2.5 | 12.69 | rectum | 3 | Present | Present | Absent | 3 | 0.8 | homogeneous | 0.7 | T3 |
| 24 | female | 84 | 1.71 | 8.26 | rectum | 4 | Present | Present | Absent | 3.5 | 1.4 | homogeneous | 1.2 | ≤T2 |
| 25 | male | 57 | 4.34 | 3.13 | left transverse | 4 | Present | Present | Present | 7.5 | 2.6 | inhomogeneous | 0.52 | T4 |
| 26 | male | 70 | 41.25 | 0.6 | rectum | 4 | Present | Present | Present | 4.6 | 1.5 | homogeneous | 0.36 | T4 |
| 27 | male | 49 | 1.73 | 4.82 | descending | 4 | Present | Present | Absent | 5.6 | 0.8 | homogeneous | 1.26 | T4 |
| 28 | female | 54 | 0.58 | 5.22 | rectum | 2 | Absent | Absent | Absent | 1.6 | 1.1 | homogeneous | 1.67 | T3 |
| 29 | female | 66 | 82.29 | 282.03 | sigmoid | 4 | Present | Present | Absent | 6.6 | 1.2 | inhomogeneous | 1.39 | T3 |
| 30 | male | 66 | 2.05 | 7.8 | rectum | 2 | Absent | Absent | Absent | 2.5 | 0.3 | homogeneous | 1.15 | T3 |
| 31 | male | 59 | 8.18 | 25.67 | rectum | 2 | Absent | Absent | Absent | 3.7 | 0.8 | homogeneous | 1.52 | T3 |
| 32 | male | 64 | 1.68 | 8.04 | rectum | 3 | Absent | Absent | Absent | 5.8 | 0.8 | homogeneous | 2.37 | ≤T2 |
| 33 | female | 50 | 0.14 | 11.34 | rectum | 3 | Absent | Absent | Absent | 4.3 | 0.6 | homogeneous | 1.97 | ≤T2 |
| 34 | male | 67 | 3.01 | 18.31 | ascending | 4 | Present | Present | Absent | 2.5 | 1.2 | inhomogeneous | 0.61 | T3 |
| 35 | male | 57 | 6.39 | 10.52 | sigmoid | 4 | Present | Present | Absent | 7.8 | 2 | inhomogeneous | 1.17 | ≤T2 |
| 36 | male | 43 | 3.86 | 6.36 | ascending | 4 | Present | Present | Present | 5 | 1.8 | homogeneous | 1 | T3 |
| 37 | female | 66 | 24.73 | 51.75 | rectum | 4 | Present | Present | Present | 4.9 | 1.3 | inhomogeneous | 1.46 | T4 |
| 38 | male | 74 | 4.28 | 13.92 | rectum | 3 | Present | Present | Absent | 4 | 0.8 | homogeneous | 1.84 | T3 |
| 39 | male | 52 | 0.53 | 13.95 | sigmoid | 2 | Absent | Absent | Absent | 2.8 | 0.4 | homogeneous | 1.12 | T3 |
| 40 | female | 74 | 3.75 | 11.58 | cecum | 4 | Present | Present | Absent | 4.5 | 1.2 | inhomogeneous | 1.33 | T4 |
| 41 | male | 38 | 1.67 | 17.79 | ascending | 4 | Present | Present | Absent | 3.9 | 1.2 | homogeneous | 1.74 | T3 |
| 42 | female | 49 | 11.38 | 89.53 | sigmoid | 4 | Present | Present | Absent | 6.8 | 0.6 | inhomogeneous | 0.82 | T4 |
| 43 | male | 71 | 3.58 | 13.78 | sigmoid | 4 | Present | Present | Present | 6.5 | 1.5 | homogeneous | 0.59 | T3 |
| 44 | female | 84 | 5.75 | 0.76 | cecum | 4 | Present | Present | Absent | 4.8 | 0.8 | homogeneous | 1.95 | T3 |
| 45 | male | 69 | 4.11 | 13.56 | rectum | 4 | Present | Present | Absent | 5.7 | 1.6 | homogeneous | 0.69 | T3 |
| 46 | male | 68 | 8.47 | 22.87 | sigmoid | 3 | Absent | Absent | Absent | 5 | 0.6 | homogeneous | 0.41 | T3 |
| 47 | female | 47 | 5.2 | 18 | descending | 4 | Present | Present | Present | 6.5 | 1.8 | inhomogeneous | 0.67 | T3 |
| 48 | male | 55 | 6.13 | 23.79 | rectum | 3 | Present | Present | Present | 6.2 | 2.5 | inhomogeneous | 0.73 | T3 |
| 49 | male | 61 | 4.56 | 28.18 | descending | 4 | Present | Absent | Absent | 6.6 | 0.4 | homogeneous | 1.23 | T3 |
| 50 | male | 63 | 0.94 | 4.75 | sigmoid | 4 | Present | Present | Absent | 3.1 | 1 | homogeneous | 1.25 | T3 |
| 51 | male | 65 | 0.56 | 8.38 | sigmoid | 3 | Absent | Absent | Absent | 3.1 | 0.4 | homogeneous | 1.09 | ≤T2 |
| 52 | female | 57 | 6.52 | 52.48 | rectum | 4 | Absent | Absent | Absent | 2.6 | 1.8 | homogeneous | 0.98 | T3 |
| 53 | female | 77 | 147.9 | 14.5 | sigmoid | 4 | Present | Present | Present | 7 | 2 | inhomogeneous | 0.73 | T4 |
| 54 | male | 51 | 23.76 | 0.6 | sigmoid | 4 | Present | Present | Absent | 10.4 | 1 | homogeneous | 0.73 | T3 |
| 55 | male | 77 | 3.86 | 50.5 | sigmoid | 4 | Absent | Absent | Absent | 3.7 | 1.7 | homogeneous | 1.54 | T3 |
| 56 | male | 53 | 3.84 | 18.92 | descending | 4 | Present | Present | Absent | 9.3 | 1.3 | inhomogeneous | 0.64 | T3 |
| 57 | male | 69 | 1.58 | 4.13 | sigmoid | 4 | Present | Present | Absent | 5 | 0.6 | homogeneous | 1.03 | T3 |
| 58 | female | 45 | 8.87 | 537.15 | ascending | 4 | Present | Present | Absent | 6.7 | 2.1 | inhomogeneous | 1.08 | T3 |
| 59 | female | 55 | 2.78 | 79.79 | sigmoid | 2 | Absent | Absent | Absent | 1.6 | 1 | homogeneous | 1.13 | ≤T2 |
| 60 | male | 59 | 0.81 | 8.37 | sigmoid | 2 | Absent | Absent | Absent | 1.2 | 0.2 | homogeneous | 0.65 | ≤T2 |
| 61 | male | 50 | 17.87 | 14.92 | right transverse | 4 | Present | Present | Absent | 4.7 | 1.1 | homogeneous | 0.83 | T4 |
| 62 | male | 71 | 2.03 | 22.45 | sigmoid | 4 | Absent | Absent | Absent | 7.2 | 1.3 | homogeneous | 1.62 | T3 |
| 63 | female | 30 | 2.72 | 182.61 | ascending | 4 | Present | Present | Absent | 5.6 | 1.4 | homogeneous | 1.08 | T4 |
| 64 | male | 54 | 2.39 | 9.82 | sigmoid | 4 | Present | Present | Absent | 4.5 | 0.8 | homogeneous | 0.96 | T3 |
| 65 | female | 61 | 2.89 | 1000 | ascending | 4 | Present | Present | Present | 1.3 | 2.8 | inhomogeneous | 1.5 | T4 |
| 66 | male | 53 | 2.23 | 7.31 | sigmoid | 3 | Present | Present | Absent | 6 | 2.1 | homogeneous | 1.32 | T3 |
| 67 | female | 62 | 6.74 | 17.79 | cecum | 4 | Present | Present | Absent | 4.3 | 1.3 | inhomogeneous | 1.23 | T3 |
| 68 | female | 70 | 0.92 | 7.85 | cecum | 4 | Present | Present | Absent | 3.4 | 0.9 | homogeneous | 1.74 | T3 |
| 69 | male | 74 | 5.72 | 5.64 | sigmoid | 4 | Present | Present | Absent | 5.1 | 0.9 | homogeneous | 0.97 | T3 |
| 70 | male | 39 | 3.25 | 25.07 | sigmoid | 4 | Present | Absent | Absent | 4.1 | 1.2 | homogeneous | 0.73 | T3 |
| 71 | female | 74 | 4.24 | 9.85 | sigmoid | 4 | Absent | Absent | Present | 4.3 | 0.9 | homogeneous | 1.12 | T3 |
| 72 | male | 51 | 40.68 | 11.64 | rectum | 2 | Absent | Absent | Absent | 4 | 0.9 | homogeneous | 1.14 | T3 |
| 73 | male | 42 | 2.95 | 9.33 | rectum | 3 | Absent | Absent | Absent | 2.6 | 1 | homogeneous | 0.72 | ≤T2 |
| 74 | female | 64 | 1.3 | 19.54 | rectum | 3 | Absent | Absent | Absent | 1.5 | 0.3 | homogeneous | 0.72 | ≤T2 |
| 75 | female | 70 | 2.47 | 17.44 | sigmoid | 3 | Absent | Absent | Absent | 2.1 | 0.4 | inhomogeneous | 1.06 | T3 |
| 76 | female | 71 | 1.16 | 12.02 | rectum | 2 | Absent | Absent | Absent | 2.7 | 1.1 | homogeneous | 1.8 | ≤T2 |
| 77 | female | 53 | 3.76 | 25.2 | rectum | 2 | Present | Absent | Absent | 5 | 1.1 | homogeneous | 1.1 | T3 |
| 78 | male | 69 | 2.73 | 15.13 | rectum | 2 | Absent | Absent | Absent | 3.5 | 0.8 | homogeneous | 1.32 | ≤T2 |
| 79 | male | 63 | 0.86 | 12.16 | cecum | 4 | Present | Present | Absent | 3.8 | 0.6 | inhomogeneous | 0.92 | T3 |
| 80 | female | 73 | 2 | 10 | ascending | 4 | Present | Present | Absent | 10.4 | 1.3 | homogeneous | 0.97 | T3 |
| 81 | female | 57 | 1.53 | 6.72 | rectum | 3 | Present | Absent | Absent | 2.8 | 0.5 | homogeneous | 1.16 | T4 |
| 82 | female | 66 | 7.2 | 30.39 | rectum | 2 | Absent | Absent | Absent | 2.6 | 0.9 | homogeneous | 1.19 | ≤T2 |
| 83 | male | 70 | 19.51 | 13.08 | sigmoid | 4 | Present | Absent | Absent | 3.4 | 11.2 | homogeneous | 0.96 | T3 |
| 84 | male | 43 | 35.05 | 2.93 | cecum | 4 | Present | Present | Absent | 4.7 | 1.2 | homogeneous | 1.13 | T3 |
| 85 | female | 77 | 0.99 | 20.56 | ascending | 4 | Absent | Absent | Absent | 2.5 | 0.9 | inhomogeneous | 0.42 | T3 |
| 86 | female | 52 | 1.17 | 78.55 | sigmoid | 4 | Present | Present | Absent | 6.7 | 0.4 | homogeneous | 1.06 | T3 |
| 87 | male | 62 | 2.4 | 6.35 | sigmoid | 4 | Present | Absent | Absent | 3.7 | 1.3 | homogeneous | 0.47 | T3 |
| 88 | female | 76 | 4.09 | 31.43 | sigmoid | 4 | Present | Present | Absent | 6.2 | 1.6 | homogeneous | 1.34 | T3 |
| 89 | male | 45 | 19.15 | 5.27 | sigmoid | 4 | Present | Present | Present | 6.7 | 1.6 | inhomogeneous | 0.85 | T3 |
| 90 | male | 49 | 2.9 | 13.39 | ascending | 4 | Present | Present | Absent | 3 | 0.8 | homogeneous | 0.81 | T3 |
| 91 | female | 60 | 3.93 | 13.49 | right transverse | 4 | Absent | Absent | Absent | 3.4 | 0.5 | homogeneous | 1.14 | T3 |
| 92 | male | 65 | 10.48 | 44.49 | sigmoid | 4 | Present | Present | Absent | 6 | 1.4 | homogeneous | 0.8 | T3 |
| 93 | female | 61 | 6.5 | 15.85 | sigmoid | 3 | Absent | Absent | Absent | 1.9 | 0.5 | homogeneous | 1.02 | T3 |
| 94 | female | 48 | 0.79 | 11.44 | cecum | 4 | Present | Absent | Absent | 4.8 | 1.4 | inhomogeneous | 0.89 | T3 |
| 95 | female | 71 | 2.22 | 11.73 | sigmoid | 3 | Present | Absent | Absent | 3 | 0.6 | homogeneous | 1.03 | ≤T2 |
| 96 | female | 69 | 5.94 | 30.76 | rectum | 4 | Present | Present | Absent | 5 | 0.8 | homogeneous | 1.24 | T3 |
| 97 | male | 48 | 24.08 | 181.5 | cecum | 4 | Present | Present | Absent | 3.4 | 1.1 | inhomogeneous | 1.13 | T3 |
| 98 | female | 66 | 52.06 | 23.84 | sigmoid | 4 | Present | Present | Absent | 6.1 | 0.9 | homogeneous | 0.9 | T3 |
| 99 | male | 55 | 1.62 | 7.08 | sigmoid | 4 | Present | Present | Absent | 14 | 0.9 | inhomogeneous | 0.77 | T3 |
| 100 | female | 58 | 365.6 | 800 | rectum | 2 | Present | Present | Absent | 4.4 | 1.5 | homogeneous | 1.54 | T3 |
| 101 | male | 53 | 4.33 | 15.68 | left transverse | 4 | Present | Present | Absent | 6 | 1.1 | inhomogeneous | 0.77 | T4 |
| 102 | female | 53 | 16.4 | 111 | rectum | 4 | Present | Absent | Absent | 4.4 | 0.7 | inhomogeneous | 1.5 | T4 |
| 103 | male | 65 | 2.81 | 3.73 | left transverse | 4 | Present | Present | Absent | 6.6 | 1.1 | inhomogeneous | 0.93 | T4 |
| 104 | female | 61 | 1.71 | 5.54 | sigmoid | 4 | Present | Present | Present | 5.3 | 1 | homogeneous | 0.92 | T4 |
| 105 | male | 59 | 4.8 | 11.62 | rectum | 3 | Present | Absent | Absent | 4 | 2.2 | homogeneous | 1.36 | T4 |
| 106 | female | 33 | 1.41 | 13.28 | left transverse | 4 | Present | Present | Absent | 4.8 | 1.3 | homogeneous | 0.77 | T4 |
| 107 | male | 53 | 35.39 | 6.63 | sigmoid | 4 | Present | Absent | Absent | 4.3 | 1 | homogeneous | 0.95 | T4 |
| 108 | male | 46 | 6.46 | 21.07 | rectum | 2 | Present | Present | Present | 10.2 | 3.4 | homogeneous | 0.54 | T3 |
| 109 | female | 38 | 1.59 | 5.6 | rectum | 4 | Absent | Absent | Absent | 4 | 0.7 | homogeneous | 0.95 | T4 |
| 110 | male | 60 | 14.25 | 0.6 | rectum | 4 | Absent | Absent | Absent | 4 | 0.9 | homogeneous | 0.84 | T4 |
| 111 | female | 49 | 2.37 | 22.41 | rectum | 3 | Absent | Absent | Absent | 3.1 | 0.6 | homogeneous | 1.18 | T3 |
| 112 | male | 79 | 3 | 30 | rectum | 3 | Absent | Absent | Absent | 2 | 1.4 | homogeneous | 0.97 | T3 |
| 113 | female | 76 | 15.74 | 17.92 | sigmoid | 4 | Present | Absent | Absent | 3.1 | 1.1 | homogeneous | 0.72 | T4 |
| 114 | male | 81 | 6.21 | 26.62 | sigmoid | 4 | Present | Present | Absent | 3.6 | 0.8 | homogeneous | 1.14 | T4 |
| 115 | female | 48 | 0.8 | 0.6 | rectum | 3 | Present | Absent | Absent | 3.7 | 0.6 | homogeneous | 0.97 | T4 |
| 116 | male | 67 | 6.72 | 13.81 | sigmoid | 1 | Absent | Absent | Absent | 0.8 | 0.4 | inhomogeneous | 0.77 | ≤T2 |
| 117 | male | 65 | 6.72 | 13.81 | rectum | 3 | Absent | Absent | Absent | 3.6 | 1.2 | homogeneous | 0.79 | T4 |
| 118 | male | 63 | 3.29 | 5.82 | sigmoid | 2 | Present | Present | Absent | 2.4 | 1 | homogeneous | 0.87 | T3 |
| 119 | female | 62 | 35.19 | 26.3 | sigmoid | 4 | Present | Present | Present | 11.7 | 1.4 | inhomogeneous | 0.61 | T4 |
| 120 | female | 50 | 4.36 | 11.35 | rectum | 4 | Present | Present | Absent | 5.1 | 0.8 | homogeneous | 0.54 | T4 |
| 121 | male | 77 | 14.7 | 11.35 | rectum | 4 | Present | Present | Absent | 5.7 | 1.5 | homogeneous | 1.08 | T4 |
| 122 | female | 77 | 2.16 | 13.15 | sigmoid | 4 | Absent | Present | Absent | 6.8 | 2.1 | inhomogeneous | 1.14 | T3 |
| 123 | female | 68 | 31.9 | 23.23 | cecum | 4 | Present | Present | Absent | 10.4 | 1 | inhomogeneous | 1.4 | T3 |
| 124 | male | 70 | 7.7 | 25.89 | rectum | 4 | Present | Absent | Absent | 3.5 | 0.5 | homogeneous | 0.55 | T3 |
| 125 | female | 80 | 3.32 | 15.75 | sigmoid | 3 | Present | Absent | Absent | 3.7 | 0.8 | homogeneous | 1.8 | ≤T2 |
| 126 | male | 48 | 4.4 | 211.3 | sigmoid | 4 | Present | Present | Absent | 7.3 | 0.9 | homogeneous | 0.79 | T3 |
| 127 | male | 55 | 1.45 | 64.03 | cecum | 4 | Present | Present | Absent | 5.4 | 1.2 | inhomogeneous | 0.62 | T3 |
| 128 | female | 74 | 47.39 | 100.5 | rectum | 4 | Present | Absent | Absent | 4.2 | 1 | homogeneous | 1.37 | T3 |
| 129 | male | 85 | 5 | 13.59 | cecum | 4 | Present | Present | Absent | 7.1 | 2 | inhomogeneous | 1.11 | T3 |
| 130 | female | 67 | 2 | 13 | sigmoid | 4 | Absent | Absent | Absent | 1.7 | 0.6 | inhomogeneous | 1.44 | ≤T2 |
| 131 | female | 64 | 3 | 10 | descending | 4 | Absent | Present | Absent | 5.7 | 0.9 | inhomogeneous | 1.25 | T3 |
| 132 | male | 52 | 0.8 | 0.6 | rectum | 4 | Present | Absent | Absent | 4.3 | 0.8 | homogeneous | 0.86 | T3 |
| 133 | female | 59 | 48.4 | 40.87 | right transverse | 4 | Present | Present | Absent | 5.4 | 0.9 | homogeneous | 1.24 | T3 |
| 134 | male | 59 | 26.63 | 45.06 | cecum | 4 | Present | Present | Absent | 11.4 | 1 | inhomogeneous | 1.02 | T4 |
| 135 | male | 52 | 6.37 | 6.74 | sigmoid | 4 | Absent | Present | Absent | 3.9 | 1 | inhomogeneous | 1.52 | T3 |
| 136 | male | 46 | 4.89 | 3.2 | sigmoid | 3 | Absent | Absent | Absent | 3.2 | 0.5 | homogeneous | 0.76 | T3 |
| 137 | male | 44 | 1.76 | 3.54 | cecum | 4 | Present | Present | Absent | 4.2 | 1.4 | homogeneous | 0.71 | T3 |
| 138 | female | 72 | 16.91 | 10.71 | sigmoid | 3 | Present | Absent | Absent | 3.6 | 0.6 | homogeneous | 0.83 | T3 |
| 139 | female | 62 | 0.6 | 7.4 | rectum | 2 | Absent | Absent | Absent | 2.1 | 0.5 | homogeneous | 0.7 | ≤T2 |
| 140 | female | 28 | 0.76 | 8.39 | left transverse | 4 | Present | Present | Absent | 8.4 | 1.1 | inhomogeneous | 1.23 | T3 |
| 141 | male | 62 | 2.3 | 12.47 | sigmoid | 4 | Absent | Absent | Absent | 4.5 | 1 | homogeneous | 0.83 | T3 |
| 142 | male | 63 | 7.32 | 9.73 | sigmoid | 2 | Present | Absent | Absent | 3.2 | 0.7 | homogeneous | 0.89 | ≤T2 |
| 143 | female | 67 | 2.45 | 4.88 | rectum | 4 | Absent | Absent | Absent | 3.8 | 0.6 | homogeneous | 1.4 | ≤T2 |
| 144 | male | 52 | 1.46 | 18.61 | rectum | 2 | Present | Absent | Absent | 5.9 | 1.2 | homogeneous | 1.02 | T3 |
| 145 | male | 61 | 7.42 | 50.48 | rectum | 4 | Present | Present | Absent | 4.5 | 0.6 | inhomogeneous | 1.25 | T3 |
| 146 | female | 71 | 10.79 | 36.26 | rectum | 2 | Absent | Present | Absent | 2.9 | 0.6 | homogeneous | 1.01 | T3 |
| 147 | male | 52 | 3.19 | 4.17 | rectum | 3 | Absent | Absent | Absent | 5.8 | 2.5 | homogeneous | 0.41 | ≤T2 |
| 148 | female | 63 | 2.57 | 0.6 | ascending | 4 | Present | Present | Absent | 2.5 | 1 | homogeneous | 1.17 | T3 |
| 149 | female | 50 | 4.68 | 6.9 | sigmoid | 2 | Absent | Absent | Absent | 4 | 0.6 | homogeneous | 1.46 | T3 |
| 150 | male | 81 | 2.73 | 8.98 | rectum | 2 | Present | Present | Absent | 4.5 | 1 | homogeneous | 0.7 | T3 |
| 151 | female | 72 | 6.32 | 10.24 | rectum | 2 | Absent | Absent | Absent | 2.9 | 1.1 | homogeneous | 1.22 | ≤T2 |
| 152 | male | 60 | 4.34 | 9.14 | descending | 4 | Present | Present | Absent | 3.5 | 1.1 | homogeneous | 0.82 | T3 |
| 153 | male | 70 | 2.75 | 5.53 | rectum | 4 | Present | Absent | Absent | 2.9 | 0.5 | homogeneous | 0.72 | T3 |
| 154 | male | 75 | 4 | 10 | sigmoid | 4 | Absent | Present | Absent | 4.7 | 0.5 | homogeneous | 1.07 | ≤T2 |
| 155 | female | 60 | 10.64 | 6.83 | sigmoid | 4 | Present | Absent | Absent | 4.1 | 0.8 | homogeneous | 0.9 | T3 |
| 156 | female | 66 | 1.43 | 20.97 | rectum | 2 | Present | Absent | Absent | 2.5 | 0.6 | homogeneous | 0.25 | ≤T2 |
| 157 | male | 47 | 1.88 | 8.46 | left transverse | 4 | Present | Present | Absent | 4.1 | 0.7 | homogeneous | 0.3 | T3 |
| 158 | female | 48 | 2.55 | 7.34 | sigmoid | 4 | Present | Absent | Absent | 2.6 | 0.7 | homogeneous | 0.13 | T3 |
| 159 | female | 47 | 0.46 | 6.42 | rectum | 2 | Absent | Absent | Absent | 1.2 | 0.5 | homogeneous | 2.24 | ≤T2 |
| 160 | female | 62 | 1.41 | 16.26 | rectum | 4 | Present | Present | Absent | 5.1 | 0.7 | homogeneous | 0.94 | T3 |
| 161 | male | 47 | 143.8 | 73.75 | rectum | 4 | Present | Present | Absent | 3.7 | 1.2 | homogeneous | 1.16 | T3 |
| 162 | female | 57 | 0.82 | 6.68 | cecum | 4 | Present | Present | Absent | 4.8 | 1 | homogeneous | 0.8 | ≤T2 |
| 163 | male | 44 | 3.07 | 7.14 | sigmoid | 2 | Present | Absent | Absent | 2.6 | 0.3 | homogeneous | 0.62 | ≤T2 |
| 164 | female | 63 | 11.5 | 10.67 | rectum | 3 | Present | Present | Absent | 4.4 | 1 | homogeneous | 0.85 | T3 |
| 165 | male | 66 | 11.77 | 18.93 | right transverse | 4 | Present | Present | Absent | 6.3 | 0.8 | inhomogeneous | 0.63 | T3 |
| 166 | female | 58 | 1.61 | 17.37 | rectum | 2 | Present | Absent | Absent | 2.8 | 0.4 | inhomogeneous | 1.39 | ≤T2 |
| 167 | male | 59 | 9.47 | 0.6 | ascending | 4 | Absent | Absent | Absent | 4.2 | 1.5 | homogeneous | 0.66 | T3 |
| 168 | female | 48 | 1.91 | 41.58 | right transverse | 4 | Present | Present | Absent | 5.1 | 0.9 | homogeneous | 0.83 | T3 |
| 169 | male | 45 | 2.98 | 22.1 | rectum | 2 | Present | Absent | Absent | 4.3 | 0.7 | homogeneous | 0.42 | T3 |
| 170 | male | 56 | 25.99 | 19.84 | descending | 4 | Present | Present | Absent | 6.5 | 0.9 | homogeneous | 0.44 | T4 |
| 171 | male | 65 | 2 | 19.07 | descending | 2 | Present | Absent | Absent | 4 | 0.6 | homogeneous | 0.84 | ≤T2 |
| 172 | male | 75 | 60.38 | 28.05 | sigmoid | 4 | Present | Present | Absent | 5.1 | 1.2 | inhomogeneous | 0.61 | T3 |
| 173 | male | 48 | 3.89 | 353.7 | left transverse | 4 | Present | Present | Absent | 6.6 | 1.1 | homogeneous | 0.99 | T4 |
| 174 | male | 58 | 8.97 | 5.53 | sigmoid | 4 | Present | Present | Absent | 6.9 | 1.7 | homogeneous | 0.74 | T3 |
| 175 | male | 59 | 1.04 | 9.52 | ascending | 4 | Present | Present | Absent | 4.4 | 1 | inhomogeneous | 1.42 | T3 |
| 176 | male | 81 | 35.01 | 15.03 | cecum | 4 | Absent | Present | Absent | 3.6 | 1 | homogeneous | 1.55 | T4 |
| 177 | female | 73 | 2.78 | 21.59 | rectum | 2 | Present | Present | Absent | 2.3 | 1.6 | homogeneous | 0.76 | ≤T2 |
| 178 | male | 62 | 3.08 | 8.87 | rectum | 4 | Absent | Present | Absent | 3.3 | 0.8 | homogeneous | 0.98 | ≤T2 |
| 179 | female | 67 | 1.06 | 2.03 | rectum | 2 | Absent | Absent | Absent | 1.4 | 0.8 | homogeneous | 1.22 | ≤T2 |
| 180 | male | 67 | 5.88 | 12.16 | sigmoid | 4 | Present | Present | Absent | 4.4 | 0.9 | homogeneous | 0.31 | T3 |
| 181 | male | 66 | 13.46 | 4.36 | sigmoid | 4 | Present | Present | Absent | 8.1 | 1.6 | homogeneous | 0.3 | T3 |
| 182 | female | 53 | 0.77 | 20.53 | sigmoid | 3 | Present | Absent | Absent | 2.6 | 0.6 | homogeneous | 2.6 | T3 |
| 183 | male | 65 | 3.21 | 23.35 | sigmoid | 4 | Present | Absent | Absent | 5 | 1 | homogeneous | 0.99 | T3 |
| 184 | female | 47 | 3.24 | 33.38 | sigmoid | 3 | Present | Present | Absent | 4.1 | 1 | homogeneous | 0.39 | T3 |
| 185 | female | 53 | 8.22 | 45.63 | sigmoid | 4 | Present | Present | Absent | 4.2 | 1.2 | inhomogeneous | 1.46 | T3 |
| 186 | female | 64 | 13.59 | 10.45 | ascending | 4 | Present | Present | Absent | 3.8 | 0.8 | homogeneous | 0.88 | T3 |
| 187 | male | 62 | 5.65 | 100.7 | ascending | 4 | Absent | Absent | Absent | 8.5 | 1.2 | inhomogeneous | 0.56 | T4 |
| 188 | female | 66 | 1.36 | 0.6 | rectum | 1 | Absent | Absent | Absent | 0.8 | 0.3 | homogeneous | 0.66 | ≤T2 |
| 189 | female | 43 | 1.25 | 9.39 | sigmoid | 4 | Present | Absent | Absent | 2.9 | 0.6 | homogeneous | 2.88 | T3 |
| 190 | female | 58 | 10.87 | 16.46 | rectum | 2 | Present | Absent | Absent | 4.1 | 1 | homogeneous | 0.63 | T3 |
| 191 | female | 63 | 1.74 | 27.73 | rectum | 3 | Absent | Absent | Absent | 3.7 | 1.2 | homogeneous | 1.09 | ≤T2 |
| 192 | male | 76 | 2.17 | 14.62 | sigmoid | 4 | Present | Present | Absent | 6.2 | 1.8 | inhomogeneous | 1.68 | T3 |
| 193 | male | 63 | 2.67 | 6.21 | rectum | 1 | Absent | Absent | Absent | 0.7 | 0.5 | homogeneous | 0.64 | ≤T2 |
| 194 | female | 62 | 8.27 | 17.78 | sigmoid | 4 | Present | Present | Absent | 4.2 | 0.5 | homogeneous | 1 | T3 |
| 195 | female | 77 | 3.01 | 9.9 | sigmoid | 3 | Present | Absent | Absent | 2.7 | 1.2 | homogeneous | 1.03 | ≤T2 |
| 196 | male | 69 | 4.46 | 40.87 | rectum | 4 | Present | Absent | Absent | 4.9 | 1.3 | homogeneous | 1.02 | ≤T2 |
| 197 | male | 55 | 2.96 | 11.44 | sigmoid | 3 | Present | Absent | Absent | 3.3 | 0.9 | homogeneous | 0.63 | T3 |
| 198 | male | 74 | 5.23 | 11.74 | cecum | 4 | Present | Present | Absent | 4.6 | 1.3 | inhomogeneous | 1.23 | T4 |
| 199 | female | 59 | 3.08 | 10 | rectum | 3 | Absent | Absent | Absent | 3.6 | 1 | homogeneous | 0.94 | ≤T2 |
| 200 | male | 65 | 2.5 | 10 | rectum | 4 | Present | Absent | Absent | 4.1 | 0.8 | homogeneous | 0.56 | T3 |
| 201 | male | 71 | 1.38 | 22.2 | right transverse | 4 | Absent | Absent | Absent | 4.2 | 1.2 | homogeneous | 0.15 | T3 |
| 202 | female | 77 | 3 | 15.06 | descending | 4 | Present | Present | Absent | 2.6 | 1 | homogeneous | 0.87 | T3 |
| 203 | male | 75 | 6.41 | 12.36 | ascending | 4 | Present | Present | Absent | 4.7 | 1.5 | inhomogeneous | 0.57 | T3 |
| 204 | male | 74 | 38.79 | 68.73 | rectum | 4 | Present | Present | Absent | 4.2 | 1.2 | homogeneous | 0.57 | T3 |
| 205 | female | 68 | 22.2 | 21.59 | right transverse | 4 | Present | Present | Absent | 3.8 | 0.6 | inhomogeneous | 1.48 | T4 |
| 206 | male | 60 | 33.6 | 15.74 | sigmoid | 4 | Present | Present | Absent | 4.8 | 0.8 | homogeneous | 0.61 | T3 |
| 207 | female | 52 | 0.85 | 7.64 | right transverse | 4 | Present | Present | Absent | 8 | 1.5 | homogeneous | 1.1 | T4 |
| 208 | male | 91 | 4.19 | 15.8 | right transverse | 4 | Present | Present | Absent | 4.4 | 0.5 | homogeneous | 1.23 | T3 |
| 209 | male | 45 | 2.52 | 5.9 | rectum | 4 | Present | Present | Absent | 3.9 | 1.2 | homogeneous | 0.94 | T3 |
| 210 | male | 66 | 3.67 | 5.96 | rectum | 4 | Present | Present | Present | 7.3 | 1.8 | inhomogeneous | 0.54 | T3 |
| 211 | male | 58 | 2.63 | 3.49 | sigmoid | 4 | Present | Present | Absent | 7.2 | 0.8 | homogeneous | 1.01 | T3 |
| 212 | male | 65 | 1.66 | 5.08 | rectum | 3 | Present | Absent | Absent | 2.5 | 1.1 | homogeneous | 0.61 | T3 |
| 213 | male | 63 | 20.87 | 2.43 | rectum | 4 | Absent | Absent | Absent | 3.1 | 0.8 | homogeneous | 0.62 | T3 |
| 214 | male | 54 | 6.9 | 1000 | ascending | 4 | Present | Present | Absent | 6.1 | 1 | inhomogeneous | 1.23 | T3 |
| 215 | male | 68 | 3.72 | 9.96 | sigmoid | 4 | Present | Absent | Absent | 2.7 | 0.9 | homogeneous | 0.79 | T3 |
| 216 | female | 47 | 1.05 | 10.62 | ascending | 4 | Present | Present | Absent | 4.6 | 1.6 | homogeneous | 1.03 | T3 |
| 217 | male | 54 | 2.12 | 12.67 | rectum | 4 | Present | Absent | Absent | 5 | 1 | homogeneous | 0.88 | T3 |
| 218 | female | 71 | 1.26 | 22 | rectum | 2 | Absent | Absent | Absent | 3.5 | 1 | homogeneous | 0.62 | ≤T2 |
| 219 | male | 55 | 1.16 | 3.75 | sigmoid | 4 | Present | Present | Present | 8.2 | 1.6 | homogeneous | 0.62 | T3 |
| 220 | female | 56 | 1.26 | 10.82 | sigmoid | 2 | Present | Absent | Absent | 2.8 | 0.6 | homogeneous | 0.35 | T3 |
| 221 | male | 56 | 3.15 | 10.76 | rectum | 4 | Present | Present | Absent | 4.3 | 1 | homogeneous | 1.33 | T3 |
| 222 | female | 63 | 4.4 | 10.82 | sigmoid | 4 | Present | Absent | Absent | 3.1 | 0.9 | homogeneous | 0.78 | T3 |
| 223 | female | 63 | 3.46 | 28.25 | sigmoid | 4 | Absent | Absent | Absent | 4.2 | 0.5 | homogeneous | 0.97 | T3 |
| 224 | male | 58 | 4.81 | 7.64 | sigmoid | 4 | Present | Present | Absent | 4.8 | 0.9 | homogeneous | 0.68 | T3 |
| 225 | female | 67 | 1.19 | 9.64 | sigmoid | 1 | Absent | Absent | Absent | 1 | 0.5 | homogeneous | 1.81 | ≤T2 |
| 226 | male | 61 | 3.73 | 11.03 | sigmoid | 4 | Present | Absent | Absent | 3.5 | 0.5 | homogeneous | 1.18 | T3 |
| 227 | male | 58 | 2.34 | 314 | ascending | 4 | Present | Present | Absent | 16.8 | 0.9 | inhomogeneous | 1.44 | T4 |
| 228 | male | 51 | 19.05 | 8.26 | sigmoid | 4 | Present | Present | Absent | 7.1 | 1.2 | inhomogeneous | 0.52 | T4 |
| 229 | male | 71 | 3.29 | 3.54 | sigmoid | 4 | Present | Absent | Absent | 7 | 1.2 | homogeneous | 0.86 | T3 |
| 230 | female | 78 | 6.45 | 4.95 | sigmoid | 4 | Present | Absent | Absent | 5.3 | 0.9 | homogeneous | 1.3 | ≤T2 |
| 231 | female | 63 | 1.27 | 6.85 | rectum | 2 | Absent | Absent | Absent | 2.1 | 0.6 | homogeneous | 1.4 | ≤T2 |
| 232 | female | 64 | 3.02 | 58.62 | sigmoid | 4 | Present | Present | Absent | 3.4 | 1.1 | homogeneous | 1.1 | ≤T2 |
| 233 | male | 57 | 18.26 | 71.38 | ascending | 4 | Present | Present | Absent | 6.7 | 1 | inhomogeneous | 0.93 | T4 |
| 234 | female | 55 | 0.5 | 5.56 | right transverse | 4 | Present | Absent | Absent | 2 | 1.1 | homogeneous | 1.59 | ≤T2 |
| 235 | female | 60 | 1.02 | 12.26 | sigmoid | 4 | Present | Absent | Absent | 5 | 0.8 | homogeneous | 0.59 | T3 |
| 236 | female | 37 | 0.91 | 12.05 | ascending | 4 | Present | Absent | Absent | 4.4 | 0.8 | homogeneous | 0.16 | ≤T2 |
| 237 | male | 73 | 92.04 | 10 | sigmoid | 4 | Present | Present | Absent | 4.9 | 1.8 | homogeneous | 0.51 | T3 |
| 238 | female | 56 | 6.05 | 14.65 | cecum | 4 | Present | Present | Absent | 5.9 | 1 | homogeneous | 1.15 | ≤T2 |
| 239 | female | 61 | 7.49 | 22.47 | ascending | 4 | Present | Present | Absent | 8 | 2.8 | inhomogeneous | 1.36 | T3 |
| 240 | female | 59 | 156.6 | 7.57 | rectum | 4 | Present | Present | Absent | 5.1 | 0.8 | homogeneous | 1.05 | T3 |
| 241 | female | 65 | 2.95 | 11.75 | descending | 4 | Present | Present | Absent | 9 | 2.5 | homogeneous | 0.11 | T3 |
| 242 | female | 42 | 2.16 | 0.6 | sigmoid | 4 | Present | Present | Absent | 3.4 | 0.9 | homogeneous | 0.81 | T3 |
| 243 | male | 80 | 3.33 | 14.48 | rectum | 4 | Absent | Absent | Absent | 2.2 | 0.6 | homogeneous | 2.49 | T3 |
| 244 | female | 75 | 11.78 | 12.24 | sigmoid | 4 | Present | Present | Absent | 8.8 | 1 | homogeneous | 1.04 | T3 |
| 245 | female | 63 | 96.25 | 38.36 | rectum | 4 | Present | Absent | Absent | 3 | 1.1 | homogeneous | 1.11 | T3 |
| 246 | male | 61 | 2.95 | 13.06 | rectum | 4 | Present | Absent | Absent | 3.1 | 1.1 | homogeneous | 1.06 | T3 |
| 247 | male | 45 | 2.18 | 11.14 | rectum | 3 | Present | Absent | Absent | 2.4 | 1.8 | homogeneous | 0.91 | ≤T2 |
| 248 | male | 52 | 1.47 | 0.6 | ascending | 3 | Absent | Absent | Absent | 3.1 | 3 | homogeneous | 0.99 | ≤T2 |
| 249 | male | 65 | 4.07 | 33.6 | descending | 1 | Absent | Absent | Absent | 1 | 0.3 | homogeneous | 0.52 | ≤T2 |
| 250 | male | 82 | 2.94 | 10.09 | ascending | 1 | Absent | Absent | Absent | 1 | 0.7 | homogeneous | 3.17 | ≤T2 |
| 251 | female | 71 | 0.67 | 12.93 | right transverse | 4 | Present | Present | Absent | 6.4 | 1.3 | inhomogeneous | 0.56 | ≤T2 |
| 252 | male | 65 | 1.19 | 8.05 | sigmoid | 2 | Absent | Absent | Absent | 1.2 | 0.3 | homogeneous | 0.35 | ≤T2 |
| 253 | female | 58 | 1.12 | 7.85 | descending | 3 | Present | Absent | Absent | 2.4 | 1 | homogeneous | 1.13 | ≤T2 |
| 254 | female | 50 | 1.3 | 14.25 | rectum | 2 | Absent | Absent | Absent | 1.9 | 1.2 | homogeneous | 1.4 | ≤T2 |
| 255 | male | 63 | 0.78 | 7.84 | rectum | 2 | Absent | Absent | Absent | 1.4 | 0.8 | homogeneous | 1.09 | ≤T2 |
| 256 | female | 53 | 3.17 | 11.84 | rectum | 1 | Absent | Absent | Absent | 1 | 0.5 | homogeneous | 0.87 | ≤T2 |
| 257 | female | 64 | 1.28 | 11.12 | rectum | 2 | Absent | Absent | Absent | 1.1 | 0.5 | homogeneous | 0.84 | ≤T2 |
| 258 | male | 83 | 2.91 | 0.6 | sigmoid | 3 | Present | Absent | Absent | 7 | 3.9 | inhomogeneous | 0.58 | ≤T2 |
| 259 | female | 66 | 2.04 | 5.3 | rectum | 2 | Absent | Absent | Absent | 4.1 | 2 | inhomogeneous | 0.71 | ≤T2 |
| 260 | male | 68 | 1.61 | 3.58 | rectum | 3 | Present | Absent | Absent | 1.9 | 1.5 | homogeneous | 1.53 | ≤T2 |
| 261 | male | 78 | 1.44 | 3.23 | ascending | 4 | Present | Absent | Absent | 9 | 1.1 | homogeneous | 0.56 | ≤T2 |
| 262 | male | 49 | 1.98 | 6.92 | sigmoid | 3 | Present | Absent | Absent | 2.9 | 2.3 | inhomogeneous | 1.08 | ≤T2 |
| 263 | male | 61 | 0.55 | 1.84 | cecum | 1 | Absent | Absent | Absent | 0.9 | 1.5 | homogeneous | 5.39 | ≤T2 |
| 264 | male | 62 | 2.92 | 22.28 | rectum | 3 | Absent | Absent | Absent | 2.2 | 0.6 | homogeneous | 1.02 | ≤T2 |
| 265 | male | 54 | 1.6 | 8.34 | cecum | 4 | Present | Present | Absent | 4.8 | 1.2 | inhomogeneous | 0.77 | ≤T2 |
| 266 | female | 37 | 2.2 | 10.39 | sigmoid | 3 | Absent | Absent | Absent | 1.4 | 1 | homogeneous | 0.93 | ≤T2 |
| 267 | female | 51 | 0.56 | 4.92 | sigmoid | 1 | Absent | Absent | Absent | 0.8 | 0.3 | homogeneous | 1.05 | ≤T2 |
| 268 | female | 48 | 1.13 | 30.59 | sigmoid | 3 | Present | Absent | Absent | 2 | 0.8 | homogeneous | 0.37 | ≤T2 |
| 269 | male | 42 | 2.37 | 0.6 | rectum | 2 | Absent | Absent | Absent | 1.7 | 0.4 | homogeneous | 0.52 | ≤T2 |
| 270 | male | 62 | 1.75 | 5.58 | sigmoid | 2 | Absent | Absent | Absent | 1.7 | 0.7 | homogeneous | 0.47 | ≤T2 |
| 271 | male | 59 | 0.55 | 6.21 | sigmoid | 1 | Absent | Absent | Absent | 1 | 0.3 | homogeneous | 0.89 | ≤T2 |
| 272 | male | 79 | 1.83 | 7.29 | sigmoid | 3 | Present | Absent | Absent | 3.3 | 1 | homogeneous | 0.86 | ≤T2 |
| 273 | female | 45 | 10.55 | 54.5 | descending | 4 | Present | Present | Absent | 5.4 | 0.9 | inhomogeneous | 1.08 | ≤T2 |
| 274 | female | 70 | 0.93 | 4.61 | descending | 3 | Absent | Absent | Absent | 2.8 | 1 | homogeneous | 0.87 | ≤T2 |
| 275 | female | 80 | 3.07 | 4.41 | rectum | 2 | Absent | Absent | Absent | 1.3 | 0.6 | homogeneous | 0.28 | ≤T2 |
| 276 | male | 56 | 4.56 | 0.6 | sigmoid | 1 | Absent | Absent | Absent | 1 | 0.2 | homogeneous | 0.68 | ≤T2 |
| 277 | female | 58 | 3.77 | 0.6 | rectum | 2 | Absent | Absent | Absent | 1.9 | 1.7 | homogeneous | 1 | ≤T2 |
| 278 | female | 53 | 2.36 | 11.63 | rectum | 3 | Absent | Absent | Absent | 1.2 | 0.6 | homogeneous | 1.14 | ≤T2 |
| 279 | male | 61 | 0.85 | 6.48 | sigmoid | 3 | Absent | Absent | Absent | 1.8 | 0.4 | homogeneous | 0.89 | ≤T2 |
| 280 | female | 72 | 5.72 | 40.73 | right transverse | 2 | Present | Absent | Absent | 1.5 | 1.1 | homogeneous | 0.66 | ≤T2 |
| 281 | female | 66 | 1.65 | 32.29 | rectum | 3 | Absent | Absent | Absent | 3.9 | 1.1 | homogeneous | 0.78 | ≤T2 |
| 282 | female | 41 | 0.13 | 3.4 | rectum | 1 | Absent | Absent | Absent | 0.8 | 0.5 | homogeneous | 0.66 | ≤T2 |
| 283 | male | 52 | 3.35 | 4.66 | cecum | 3 | Absent | Absent | Absent | 4.8 | 1.6 | inhomogeneous | 0.48 | ≤T2 |
| 284 | female | 62 | 2.37 | 8.19 | rectum | 3 | Present | Absent | Absent | 2.5 | 1.2 | inhomogeneous | 0.77 | ≤T2 |
| 285 | male | 60 | 0.21 | 3.76 | rectum | 2 | Absent | Absent | Absent | 1.3 | 1 | homogeneous | 2.01 | ≤T2 |
| 286 | male | 60 | 1.66 | 12.31 | rectum | 2 | Absent | Absent | Absent | 1.2 | 0.6 | homogeneous | 0.74 | ≤T2 |
| 287 | female | 61 | 0.5 | 3.76 | sigmoid | 2 | Absent | Absent | Absent | 3 | 0.5 | homogeneous | 1.14 | ≤T2 |
| 288 | female | 63 | 1.86 | 0.6 | rectum | 1 | Absent | Absent | Absent | 0.8 | 0.2 | homogeneous | 0.46 | ≤T2 |
| 289 | female | 49 | 1.39 | 0.6 | rectum | 2 | Absent | Absent | Absent | 1.6 | 0.8 | homogeneous | 0.82 | ≤T2 |
| 290 | male | 67 | 0.9 | 9.03 | sigmoid | 3 | Absent | Absent | Absent | 1.3 | 1.1 | homogeneous | 1.02 | ≤T2 |
| 291 | male | 62 | 0.74 | 5.27 | rectum | 4 | Absent | Absent | Absent | 5 | 0.7 | homogeneous | 0.95 | ≤T2 |
| 292 | male | 73 | 49.68 | 30.26 | ascending | 4 | Present | Absent | Absent | 7.3 | 1 | inhomogeneous | 1.77 | ≤T2 |
| 293 | male | 58 | 1.55 | 10.71 | sigmoid | 3 | Absent | Absent | Absent | 1.2 | 0.4 | homogeneous | 0.89 | ≤T2 |
| 294 | female | 74 | 2.37 | 12.95 | rectum | 2 | Absent | Absent | Absent | 2 | 0.5 | homogeneous | 0.66 | ≤T2 |
| 295 | female | 68 | 1.55 | 10.71 | rectum | 2 | Present | Absent | Absent | 2.5 | 0.8 | homogeneous | 0.9 | ≤T2 |
| 296 | male | 62 | 1.45 | 8.73 | rectum | 2 | Present | Absent | Absent | 3.6 | 1 | homogeneous | 0.83 | ≤T2 |
| 297 | female | 63 | 2.37 | 25.49 | rectum | 2 | Absent | Absent | Absent | 4.1 | 0.6 | homogeneous | 0.34 | ≤T2 |
| 298 | male | 53 | 2.1 | 11.13 | rectum | 3 | Absent | Absent | Absent | 3.9 | 1 | homogeneous | 0.54 | ≤T2 |
| 299 | male | 62 | 2.83 | 8.25 | rectum | 3 | Absent | Absent | Absent | 2.3 | 0.6 | homogeneous | 0.77 | ≤T2 |
| 300 | male | 59 | 3.56 | 14.81 | rectum | 2 | Absent | Absent | Absent | 2.2 | 0.6 | homogeneous | 0.58 | ≤T2 |
| 301 | female | 59 | 1.05 | 5.19 | descending | 4 | Present | Absent | Absent | 2.5 | 0.5 | homogeneous | 0.87 | ≤T2 |
| 302 | male | 66 | 3.66 | 5.66 | rectum | 3 | Present | Absent | Absent | 3.1 | 0.8 | inhomogeneous | 1.16 | ≤T2 |
| 303 | male | 55 | 1.07 | 3.1 | sigmoid | 4 | Absent | Absent | Absent | 3.2 | 0.6 | homogeneous | 1.65 | ≤T2 |
| 304 | female | 47 | 1.5 | 3 | rectum | 2 | Present | Absent | Absent | 2.3 | 1 | homogeneous | 1.2 | ≤T2 |
| 305 | male | 76 | 1.45 | 6.45 | rectum | 3 | Present | Absent | Absent | 1.6 | 0.4 | homogeneous | 0.92 | ≤T2 |
| 306 | female | 65 | 4.25 | 4.87 | rectum | 3 | Absent | Absent | Absent | 5.3 | 1 | homogeneous | 0.65 | ≤T2 |
| 307 | male | 56 | 1.04 | 5.63 | rectum | 2 | Absent | Absent | Absent | 2.9 | 0.4 | homogeneous | 0.4 | ≤T2 |
| 308 | male | 60 | 2.41 | 6.09 | rectum | 3 | Present | Absent | Absent | 5.9 | 3 | homogeneous | 0.54 | ≤T2 |
| 309 | male | 58 | 3.9 | 27.66 | rectum | 3 | Absent | Absent | Absent | 2.4 | 0.4 | homogeneous | 0.87 | ≤T2 |
| 310 | male | 60 | 0.56 | 4.19 | sigmoid | 3 | Present | Absent | Absent | 3.3 | 0.6 | homogeneous | 1.08 | ≤T2 |
| 311 | male | 39 | 1.03 | 7.95 | rectum | 3 | Absent | Absent | Absent | 2 | 1.3 | homogeneous | 0.88 | ≤T2 |
| 312 | female | 55 | 0.87 | 17.03 | rectum | 2 | Absent | Absent | Absent | 2 | 0.9 | homogeneous | 1.03 | ≤T2 |
| 313 | female | 69 | 2.62 | 9.67 | rectum | 3 | Present | Absent | Absent | 3.2 | 1.6 | homogeneous | 0.61 | ≤T2 |
| 314 | male | 80 | 3.46 | 5.34 | sigmoid | 3 | Present | Present | Absent | 6 | 2.9 | homogeneous | 0.38 | ≤T2 |
| 315 | male | 71 | 2.01 | 7.69 | rectum | 3 | Present | Absent | Absent | 2.5 | 1 | homogeneous | 0.71 | ≤T2 |
| 316 | male | 53 | 3.15 | 10.35 | rectum | 2 | Present | Absent | Absent | 2.3 | 0.9 | homogeneous | 0.87 | ≤T2 |
| 317 | male | 79 | 1.76 | 6.69 | rectum | 2 | Absent | Absent | Absent | 2.3 | 0.5 | homogeneous | 0.72 | ≤T2 |
| 318 | male | 47 | 0.57 | 3.56 | cecum | 3 | Absent | Absent | Absent | 3.8 | 1 | homogeneous | 1.15 | ≤T2 |
| 319 | male | 53 | 2.27 | 13.8 | rectum | 2 | Present | Absent | Absent | 2.3 | 0.7 | homogeneous | 0.63 | ≤T2 |
| 320 | female | 55 | 1.43 | 13.18 | rectum | 3 | Present | Present | Absent | 2.2 | 1.2 | homogeneous | 0.69 | ≤T2 |
| 321 | female | 47 | 0.48 | 6.49 | rectum | 3 | Absent | Absent | Absent | 3 | 1 | homogeneous | 0.89 | ≤T2 |
| 322 | male | 55 | 3.73 | 34.38 | sigmoid | 3 | Present | Present | Absent | 4.5 | 3.1 | inhomogeneous | 0.84 | ≤T2 |
| 323 | female | 67 | 7.85 | 0.6 | rectum | 3 | Present | Absent | Absent | 2.2 | 0.7 | homogeneous | 0.87 | ≤T2 |
| 324 | male | 72 | 8.06 | 6.58 | rectum | 3 | Present | Absent | Absent | 1.8 | 0.7 | homogeneous | 0.83 | ≤T2 |
| 325 | male | 72 | 9.18 | 4.81 | rectum | 3 | Present | Absent | Absent | 4.2 | 1.1 | homogeneous | 0.58 | ≤T2 |
| 326 | female | 63 | 0.9 | 11.9 | rectum | 2 | Absent | Absent | Absent | 4.3 | 1 | homogeneous | 1.31 | ≤T2 |
| 327 | male | 45 | 2.18 | 11.14 | rectum | 3 | Present | Absent | Absent | 2.4 | 1.8 | homogeneous | 0.91 | ≤T2 |
| 328 | male | 52 | 1.47 | 0.6 | ascending | 3 | Absent | Absent | Absent | 3.1 | 3 | homogeneous | 0.99 | ≤T2 |
| 329 | male | 65 | 4.07 | 33.6 | descending | 1 | Absent | Absent | Absent | 1 | 0.3 | homogeneous | 0.52 | ≤T2 |
| 330 | male | 82 | 2.94 | 10.09 | ascending | 1 | Absent | Absent | Absent | 1 | 0.7 | homogeneous | 3.17 | ≤T2 |
| 331 | female | 71 | 0.67 | 12.93 | right transverse | 4 | Present | Present | Absent | 6.4 | 1.3 | inhomogeneous | 0.56 | ≤T2 |
| 332 | male | 65 | 1.19 | 8.05 | sigmoid | 2 | Absent | Absent | Absent | 1.2 | 0.3 | homogeneous | 0.35 | ≤T2 |
| 333 | female | 58 | 1.12 | 7.85 | descending | 3 | Present | Absent | Absent | 2.4 | 1 | homogeneous | 1.13 | ≤T2 |
| 334 | female | 50 | 1.3 | 14.25 | rectum | 2 | Absent | Absent | Absent | 1.9 | 1.2 | homogeneous | 1.4 | ≤T2 |
| 335 | male | 63 | 0.78 | 7.84 | rectum | 2 | Absent | Absent | Absent | 1.4 | 0.8 | homogeneous | 1.09 | ≤T2 |
| 336 | female | 53 | 3.17 | 11.84 | rectum | 1 | Absent | Absent | Absent | 1 | 0.5 | homogeneous | 0.87 | ≤T2 |
| 337 | male | 71 | 3.03 | 10.59 | sigmoid | 3 | Absent | Absent | Absent | 5.2 | 3.8 | homogeneous | 0.53 | ≤T2 |
| 338 | female | 62 | 11.7 | 17.18 | rectum | 3 | Present | Absent | Absent | 1.8 | 0.9 | homogeneous | 1.51 | ≤T2 |
| 339 | female | 62 | 4.01 | 7.11 | rectum | 3 | Present | Absent | Absent | 3.1 | 0.6 | homogeneous | 0.99 | ≤T2 |
| 340 | male | 79 | 2.58 | 21.01 | rectum | 3 | Absent | Absent | Absent | 2.6 | 0.8 | homogeneous | 0.92 | ≤T2 |
| 341 | male | 59 | 13.56 | 14.24 | rectum | 4 | Absent | Absent | Absent | 6.5 | 1.3 | homogeneous | 0.73 | ≤T2 |
| 342 | male | 68 | 3.37 | 9.6 | rectum | 3 | Absent | Absent | Absent | 4.1 | 3.6 | homogeneous | 0.77 | ≤T2 |
| 343 | male | 91 | 2.69 | 15.55 | descending | 3 | Present | Absent | Absent | 5.8 | 11.4 | homogeneous | 1.24 | ≤T2 |
| 344 | male | 58 | 5.04 | 52.36 | rectum | 2 | Present | Present | Absent | 2.8 | 0.5 | homogeneous | 1.29 | ≤T2 |
| 345 | male | 68 | 1.08 | 10.5 | sigmoid | 3 | Absent | Absent | Absent | 3.7 | 1.3 | homogeneous | 0.45 | ≤T2 |
| 346 | male | 66 | 2.63 | 5.43 | rectum | 3 | Present | Absent | Absent | 2.5 | 1.6 | homogeneous | 0.72 | ≤T2 |
| 347 | male | 65 | 4.61 | 16.99 | sigmoid | 4 | Present | Absent | Absent | 3 | 1 | homogeneous | 0.82 | T4 |
| 348 | male | 66 | 4.42 | 9.66 | sigmoid | 4 | Present | Absent | Absent | 3.4 | 1 | homogeneous | 0.76 | T4 |
| 349 | male | 44 | 3.02 | 25.82 | cecum | 4 | Present | Present | Absent | 5.7 | 1.7 | inhomogeneous | 1.01 | T4 |
| 350 | female | 83 | 3.99 | 5.29 | cecum | 4 | Present | Present | Present | 6 | 1.3 | homogeneous | 0.59 | T4 |
| 351 | female | 67 | 127 | 13.44 | rectum | 4 | Present | Present | Present | 5.7 | 1.5 | inhomogeneous | 0.82 | T4 |
| 352 | female | 38 | 6.62 | 8.03 | sigmoid | 4 | Present | Present | Present | 13 | 1.3 | homogeneous | 0.31 | T4 |
| 353 | male | 75 | 1000 | 1000 | left transverse | 4 | Present | Present | Absent | 8.6 | 2 | inhomogeneous | 0.68 | T4 |
| 354 | male | 77 | 7.95 | 468.7 | sigmoid | 4 | Absent | Absent | Absent | 4.1 | 1.3 | homogeneous | 0.52 | T4 |
| 355 | male | 59 | 0.86 | 7.13 | right transverse | 4 | Present | Present | Absent | 4.2 | 1 | homogeneous | 0.68 | T4 |
| 356 | female | 52 | 3.48 | 37.05 | right transverse | 4 | Present | Present | Absent | 3.8 | 0.8 | homogeneous | 0.6 | T4 |
| 357 | female | 52 | 154.4 | 468.2 | left transverse | 4 | Present | Present | Present | 8 | 7.5 | inhomogeneous | 0.3 | T4 |
| 358 | female | 52 | 2.98 | 29.5 | sigmoid | 4 | Present | Present | Absent | 6.4 | 1.3 | inhomogeneous | 0.32 | T4 |
| 359 | male | 49 | 159.1 | 222.4 | rectum | 4 | Present | Present | Absent | 3.5 | 1.9 | homogeneous | 0.5 | T4 |
| 360 | male | 63 | 2.17 | 6.9 | sigmoid | 4 | Present | Absent | Absent | 3.8 | 1 | homogeneous | 0.21 | T4 |
| 361 | male | 53 | 2.5 | 10.5 | cecum | 4 | Present | Present | Absent | 4.6 | 1.2 | homogeneous | 0.19 | T4 |
| 362 | male | 72 | 7.66 | 780.6 | ascending | 4 | Present | Present | Absent | 10.7 | 1.6 | inhomogeneous | 0.79 | T4 |
| 363 | male | 63 | 3.4 | 26.33 | descending | 4 | Present | Absent | Absent | 6.1 | 1.3 | homogeneous | 1.12 | T4 |
| 364 | male | 42 | 12.63 | 2.4 | rectum | 3 | Present | Present | Absent | 3.4 | 1.6 | inhomogeneous | 0.35 | T4 |
| 365 | male | 55 | 4.14 | 9.27 | sigmoid | 4 | Present | Present | Present | 7.3 | 1.6 | homogeneous | 0.42 | T4 |
| 366 | female | 93 | 5.01 | 22.18 | right transverse | 4 | Present | Present | Present | 3.1 | 1.3 | inhomogeneous | 0.28 | T4 |
| 367 | male | 54 | 19.92 | 123.8 | cecum | 4 | Present | Present | Present | 9.8 | 3.4 | inhomogeneous | 0.69 | T4 |
| 368 | male | 51 | 74 | 117.6 | sigmoid | 4 | Present | Present | Present | 11.6 | 1.8 | inhomogeneous | 0.05 | T4 |
| 369 | male | 66 | 4.53 | 6.69 | sigmoid | 4 | Present | Present | Absent | 9 | 2.9 | inhomogeneous | 0.54 | T4 |
| 370 | male | 61 | 12.67 | 30.8 | right transverse | 4 | Present | Absent | Absent | 3.2 | 0.8 | homogeneous | 0.64 | T4 |
| 371 | male | 70 | 15.05 | 37.94 | descending | 3 | Present | Present | Present | 5.3 | 1.6 | inhomogeneous | 0.46 | T4 |
| 372 | male | 46 | 21.25 | 37 | descending | 4 | Present | Present | Absent | 7.3 | 2.8 | inhomogeneous | 0.55 | T4 |
| 373 | female | 53 | 12.01 | 126.98 | ascending | 4 | Present | Present | Absent | 5.4 | 1.2 | inhomogeneous | 0.47 | T4 |
| 374 | female | 56 | 5.71 | 17.39 | sigmoid | 4 | Present | Present | Present | 5.1 | 0.6 | homogeneous | 0.29 | T4 |
| 375 | male | 44 | 2.22 | 10.38 | right transverse | 4 | Present | Present | Present | 19.1 | 1.3 | inhomogeneous | 0.76 | T4 |
| 376 | male | 59 | 5.2 | 4.53 | descending | 4 | Present | Absent | Absent | 3.2 | 1 | homogeneous | 0.39 | T4 |
| 377 | male | 56 | 7.77 | 7.34 | sigmoid | 4 | Present | Present | Absent | 8.2 | 2.5 | inhomogeneous | 0.57 | T4 |
| 378 | female | 29 | 1.33 | 10.89 | descending | 4 | Present | Present | Absent | 2.6 | 1 | homogeneous | 0.58 | T4 |
| 379 | female | 75 | 1.39 | 14.63 | rectum | 4 | Present | Present | Absent | 3.7 | 11 | homogeneous | 1.26 | T4 |
| 380 | female | 66 | 29.87 | 800 | ascending | 4 | Present | Present | Absent | 11.4 | 2.5 | inhomogeneous | 1.33 | T4 |
| 381 | female | 58 | 3.29 | 62.08 | rectum | 3 | Present | Present | Absent | 3 | 10.2 | homogeneous | 0.51 | T4 |
| 382 | female | 54 | 8.26 | 43.86 | sigmoid | 4 | Present | Present | Absent | 4.3 | 1 | homogeneous | 0.89 | T4 |
| 383 | male | 76 | 10.5 | 45.29 | ascending | 4 | Present | Present | Absent | 6.9 | 1.7 | inhomogeneous | 0.84 | T4 |
| 384 | male | 45 | 1.88 | 10.04 | sigmoid | 4 | Present | Present | Absent | 6.9 | 1.1 | inhomogeneous | 1.39 | T4 |
| 385 | female | 68 | 4.12 | 9.18 | right transverse | 4 | Present | Present | Present | 13.2 | 3.2 | inhomogeneous | 0.37 | T4 |
| 386 | male | 66 | 25.17 | 25.08 | sigmoid | 4 | Present | Present | Absent | 4.6 | 1.6 | inhomogeneous | 0.79 | T4 |
| 387 | female | 51 | 10.55 | 34.19 | sigmoid | 4 | Present | Present | Absent | 6 | 1.3 | homogeneous | 0.7 | T4 |
| 388 | male | 74 | 3.62 | 18.62 | cecum | 4 | Present | Present | Absent | 3.2 | 1.2 | inhomogeneous | 1.19 | T4 |
| 389 | male | 66 | 29.46 | 27.42 | cecum | 4 | Present | Present | Present | 5.4 | 1.4 | inhomogeneous | 0.57 | T4 |
| 390 | male | 61 | 30 | 50 | sigmoid | 4 | Present | Present | Absent | 3.7 | 1 | homogeneous | 1.36 | T4 |
| 391 | male | 46 | 1.46 | 6.27 | sigmoid | 4 | Present | Present | Present | 6.8 | 1.6 | homogeneous | 0.89 | T4 |
| 392 | male | 72 | 6.34 | 4.78 | sigmoid | 4 | Present | Present | Present | 7.1 | 1.6 | inhomogeneous | 0.66 | T4 |
| 393 | female | 34 | 0.7 | 14.94 | ascending | 4 | Present | Present | Absent | 4.8 | 0.9 | homogeneous | 0.38 | T4 |
| 394 | female | 63 | 18.7 | 18.94 | cecum | 4 | Present | Present | Present | 7.7 | 2 | inhomogeneous | 1.03 | T4 |
| 395 | female | 59 | 5.17 | 14.01 | right transverse | 4 | Present | Present | Absent | 7.4 | 1.5 | homogeneous | 1.14 | T4 |
| 396 | male | 52 | 4.63 | 34.86 | cecum | 4 | Present | Present | Absent | 6.1 | 1.4 | inhomogeneous | 0.52 | T4 |
| 397 | female | 69 | 2.58 | 47.06 | sigmoid | 4 | Present | Present | Present | 6.2 | 2.1 | inhomogeneous | 0.92 | T4 |
| 398 | male | 67 | 2.35 | 14.09 | sigmoid | 4 | Present | Present | Absent | 9 | 0.9 | homogeneous | 1.21 | T4 |
| 399 | male | 51 | 115 | 0.6 | left transverse | 4 | Present | Present | Absent | 5.5 | 0.9 | homogeneous | 1.17 | T4 |
| 400 | male | 65 | 26.6 | 8.44 | descending | 4 | Present | Present | Absent | 6.3 | 1.3 | homogeneous | 0.96 | T4 |
| 401 | male | 55 | 2.46 | 5.03 | rectum | 4 | Present | Present | Absent | 7.5 | 1.6 | inhomogeneous | 0.85 | T4 |
| 402 | male | 64 | 290.6 | 906.3 | left transverse | 4 | Present | Present | Absent | 3.6 | 0.8 | inhomogeneous | 0.61 | T4 |
| 403 | female | 66 | 117.6 | 85.74 | sigmoid | 4 | Present | Present | Absent | 5.1 | 1.2 | homogeneous | 1.56 | T4 |
| 404 | male | 66 | 453.2 | 1000 | ascending | 4 | Present | Present | Absent | 8 | 1 | inhomogeneous | 0.41 | T4 |
| 405 | female | 56 | 30 | 80 | ascending | 4 | Present | Present | Absent | 6.8 | 2.3 | inhomogeneous | 0.36 | T4 |
| 406 | male | 82 | 6.99 | 25.9 | cecum | 4 | Present | Present | Present | 4.5 | 1.8 | inhomogeneous | 0.86 | T4 |
| 407 | male | 64 | 232.52 | 361.61 | descending | 4 | Present | Present | Absent | 5.3 | 1.1 | inhomogeneous | 0.76 | T4 |
| 408 | female | 80 | 58.87 | 800 | right transverse | 4 | Present | Present | Absent | 6.1 | 0.9 | inhomogeneous | 0.9 | T4 |
| 409 | female | 49 | 16.12 | 127.9 | cecum | 4 | Present | Present | Absent | 3.3 | 1.4 | homogeneous | 0.74 | T4 |
| 410 | female | 48 | 59.95 | 291.41 | left transverse | 4 | Present | Present | Absent | 5 | 1.2 | homogeneous | 0.58 | T4 |
| 411 | female | 52 | 2.06 | 12.08 | cecum | 4 | Present | Present | Present | 5.5 | 2.1 | inhomogeneous | 1.3 | T4 |
| 412 | male | 59 | 6.31 | 20.05 | rectum | 4 | Present | Absent | Absent | 3.1 | 0.9 | homogeneous | 0.95 | T4 |
| 413 | female | 69 | 1000 | 31.25 | sigmoid | 4 | Present | Present | Absent | 4.2 | 1.4 | inhomogeneous | 0.46 | T4 |
| 414 | male | 46 | 2.47 | 19.63 | ascending | 4 | Present | Present | Absent | 2.7 | 1.4 | homogeneous | 0.9 | T4 |
| 415 | female | 67 | 2.24 | 21.18 | sigmoid | 4 | Present | Absent | Absent | 2.7 | 1 | homogeneous | 0.82 | T4 |
| 416 | male | 58 | 1.63 | 5.7 | rectum | 4 | Present | Present | Absent | 5 | 3 | homogeneous | 0.77 | T4 |
| 417 | female | 58 | 3.73 | 22.69 | ascending | 4 | Present | Present | Present | 9.9 | 2 | inhomogeneous | 0.53 | T4 |
| 418 | female | 58 | 1.23 | 12.96 | ascending | 4 | Present | Present | Absent | 4.4 | 1.7 | inhomogeneous | 0.55 | T4 |
| 419 | female | 70 | 3.13 | 5.96 | ascending | 4 | Present | Present | Absent | 4.3 | 1.1 | homogeneous | 1.01 | T4 |
| 420 | male | 78 | 5.2 | 4.69 | rectum | 3 | Present | Absent | Absent | 2.4 | 0.8 | homogeneous | 0.82 | T3 |
| 421 | female | 46 | 1.02 | 34.88 | ascending | 4 | Present | Present | Absent | 5.2 | 2.3 | inhomogeneous | 0.85 | T4 |
| 422 | female | 53 | 0.5 | 2.39 | right transverse | 4 | Present | Present | Absent | 3.9 | 0.4 | homogeneous | 1.03 | T3 |
| 423 | male | 72 | 4.69 | 6.98 | ascending | 4 | Present | Present | Absent | 4.6 | 1.1 | homogeneous | 1.17 | T3 |
| 424 | male | 58 | 1.55 | 10.71 | cecum | 4 | Present | Absent | Absent | 5 | 1.2 | inhomogeneous | 1.38 | ≤T2 |
| 425 | male | 59 | 0.4 | 5.78 | rectum | 4 | Present | Present | Absent | 2.6 | 1.2 | inhomogeneous | 2.9 | T3 |
| 426 | female | 58 | 1.35 | 15 | sigmoid | 4 | Present | Absent | Absent | 3 | 0.7 | homogeneous | 0.43 | T3 |
| 427 | female | 77 | 2.39 | 3.42 | descending | 4 | Present | Present | Absent | 9.5 | 0.9 | homogeneous | 0.76 | T3 |
| 428 | male | 63 | 0.29 | 4.16 | descending | 3 | Present | Absent | Absent | 1.8 | 1.3 | homogeneous | 1.04 | ≤T2 |
| 429 | male | 51 | 23.76 | 0.6 | rectum | 4 | Present | Present | Absent | 10.4 | 1 | homogeneous | 0.73 | T3 |
| 430 | male | 77 | 3.86 | 50.5 | rectum | 4 | Absent | Absent | Absent | 3.7 | 1.7 | homogeneous | 1.54 | T3 |
| 431 | female | 54 | 2.86 | 3.24 | sigmoid | 4 | Present | Present | Absent | 6.6 | 1 | homogeneous | 0.93 | T3 |
| 432 | male | 53 | 3.84 | 18.92 | descending | 4 | Present | Present | Absent | 9.3 | 1.3 | inhomogeneous | 0.64 | T3 |
| 433 | male | 69 | 1.58 | 4.13 | rectum | 4 | Present | Present | Absent | 5 | 0.6 | homogeneous | 1.03 | T3 |
| 434 | female | 38 | 1.3 | 13.86 | rectum | 4 | Present | Absent | Absent | 3.7 | 1.1 | homogeneous | 0.94 | T3 |
| 435 | male | 70 | 3.69 | 11.37 | left transverse | 4 | Present | Present | Absent | 9.6 | 1 | homogeneous | 0.57 | T3 |
| 436 | male | 73 | 5.42 | 4.5 | rectum | 3 | Present | Absent | Absent | 3.2 | 0.8 | homogeneous | 0.84 | T3 |
| 437 | male | 62 | 1.83 | 8.92 | cecum | 4 | Present | Present | Absent | 6.3 | 1.6 | homogeneous | 1.03 | T3 |
| 438 | female | 60 | 9.55 | 80.64 | right transverse | 4 | Present | Present | Present | 15.4 | 3.1 | inhomogeneous | 0.7 | T3 |
| 439 | male | 64 | 2.13 | 24.26 | right transverse | 4 | Absent | Absent | Absent | 2.5 | 0.6 | homogeneous | 1.14 | ≤T2 |
| 440 | male | 68 | 1.73 | 4.41 | sigmoid | 3 | Present | Present | Absent | 5.2 | 3.4 | inhomogeneous | 0.53 | T3 |
| 441 | female | 62 | 2.29 | 20.02 | sigmoid | 3 | Absent | Absent | Absent | 2.1 | 0.8 | homogeneous | 0.84 | ≤T2 |
| 442 | male | 51 | 5.66 | 13.91 | sigmoid | 3 | Absent | Absent | Absent | 3.1 | 1.6 | homogeneous | 1.42 | ≤T2 |
| 443 | male | 58 | 30.61 | 23.28 | rectum | 4 | Present | Present | Absent | 3.4 | 1 | homogeneous | 0.92 | T3 |
| 444 | female | 69 | 4.08 | 114 | rectum | 4 | Present | Present | Absent | 3.2 | 1.1 | homogeneous | 1.11 | T3 |
| 445 | male | 49 | 4.12 | 4.5 | rectum | 4 | Present | Absent | Absent | 2.9 | 0.8 | homogeneous | 0.83 | T3 |
| 446 | male | 49 | 1.98 | 8.02 | rectum | 4 | Present | Present | Absent | 7.1 | 2 | homogeneous | 0.54 | T3 |
| 447 | female | 53 | 22.29 | 144.2 | rectum | 4 | Present | Absent | Absent | 4.1 | 1.2 | homogeneous | 0.85 | T3 |
| 448 | male | 59 | 0.31 | 4.16 | rectum | 4 | Present | Absent | Absent | 3.8 | 0.8 | homogeneous | 2.36 | T3 |
| 449 | male | 60 | 2.34 | 20.82 | rectum | 4 | Present | Absent | Absent | 4 | 1.3 | homogeneous | 1.22 | T3 |
| 450 | male | 60 | 2.05 | 37.65 | left transverse | 4 | Present | Absent | Absent | 3.2 | 1.1 | inhomogeneous | 0.57 | T3 |
| 451 | female | 69 | 0.99 | 13.73 | rectum | 4 | Present | Present | Absent | 2.1 | 1 | homogeneous | 1.18 | ≤T2 |
| 452 | female | 64 | 40.86 | 8.68 | rectum | 4 | Present | Present | Absent | 3.4 | 0.6 | homogeneous | 0.71 | T3 |
| 453 | female | 52 | 3.82 | 4.83 | rectum | 4 | Present | Present | Absent | 6 | 0.6 | homogeneous | 1.17 | T3 |
| 454 | female | 49 | 16.12 | 127.9 | cecum | 4 | Present | Absent | Absent | 3.9 | 1.3 | homogeneous | 0.93 | T4 |
| 455 | female | 61 | 1.32 | 0.64 | rectum | 1 | Absent | Absent | Absent | 1 | 0.3 | homogeneous | 0.48 | T3 |
| 456 | female | 61 | 24.56 | 33 | right transverse | 4 | Present | Present | Absent | 8.7 | 1 | homogeneous | 1.18 | T3 |
| 457 | female | 59 | 19.7 | 39.6 | rectum | 2 | Present | Absent | Absent | 4 | 0.8 | homogeneous | 1.15 | T3 |
| 458 | female | 61 | 1.02 | 2.23 | rectum | 3 | Present | Absent | Absent | 2.8 | 0.4 | homogeneous | 0.73 | ≤T2 |
| 459 | male | 52 | 1.06 | 3.54 | rectum | 3 | Present | Absent | Absent | 2.6 | 1 | homogeneous | 1.19 | ≤T2 |
| 460 | male | 53 | 22.82 | 15.13 | descending | 4 | Present | Present | Absent | 6.9 | 1.2 | inhomogeneous | 0.99 | T3 |
| 461 | male | 53 | 14.18 | 13.48 | sigmoid | 4 | Present | Absent | Absent | 3.5 | 0.8 | homogeneous | 0.61 | T3 |
| 462 | female | 76 | 26.69 | 11.91 | rectum | 2 | Present | Present | Absent | 4.9 | 0.6 | homogeneous | 0.99 | T3 |
| 463 | male | 46 | 4.89 | 3.2 | sigmoid | 3 | Absent | Absent | Absent | 3.2 | 0.5 | homogeneous | 0.76 | T3 |
| 464 | male | 44 | 1.76 | 3.54 | cecum | 4 | Present | Present | Absent | 4.2 | 1.4 | homogeneous | 0.71 | T3 |
| 465 | female | 72 | 16.91 | 10.71 | sigmoid | 3 | Present | Absent | Absent | 3.6 | 0.6 | homogeneous | 0.83 | T3 |
| 466 | female | 62 | 0.6 | 7.4 | rectum | 2 | Absent | Absent | Absent | 2.1 | 0.5 | homogeneous | 0.7 | ≤T2 |
| 467 | female | 28 | 0.76 | 8.39 | left transverse | 4 | Present | Present | Absent | 8.4 | 1.1 | inhomogeneous | 1.23 | T3 |
| 468 | male | 62 | 2.3 | 12.47 | sigmoid | 4 | Absent | Absent | Absent | 4.5 | 1 | homogeneous | 0.83 | T3 |
| 469 | male | 63 | 7.32 | 9.73 | sigmoid | 2 | Present | Absent | Absent | 3.2 | 0.7 | homogeneous | 0.89 | ≤T2 |
| 470 | female | 67 | 2.45 | 4.88 | rectum | 4 | Absent | Absent | Absent | 3.8 | 0.6 | homogeneous | 1.4 | ≤T2 |
| 471 | male | 52 | 1.46 | 18.61 | rectum | 2 | Present | Absent | Absent | 5.9 | 1.2 | homogeneous | 1.02 | T3 |
| 472 | male | 61 | 7.42 | 50.48 | rectum | 4 | Present | Present | Absent | 4.5 | 0.6 | inhomogeneous | 1.25 | T3 |
| 473 | female | 71 | 10.79 | 36.26 | rectum | 2 | Absent | Present | Absent | 2.9 | 0.6 | homogeneous | 1.01 | T3 |
| 474 | male | 52 | 3.19 | 4.17 | rectum | 3 | Absent | Absent | Absent | 5.8 | 2.5 | homogeneous | 0.41 | ≤T2 |
| 475 | female | 63 | 2.57 | 0.6 | ascending | 4 | Present | Present | Absent | 2.5 | 1 | homogeneous | 1.17 | T3 |
| 476 | female | 50 | 4.68 | 6.9 | sigmoid | 2 | Absent | Absent | Absent | 4 | 0.6 | homogeneous | 1.46 | T3 |
| 477 | male | 81 | 2.73 | 8.98 | rectum | 2 | Present | Present | Absent | 4.5 | 1 | homogeneous | 0.7 | T3 |
| 478 | female | 72 | 6.32 | 10.24 | rectum | 2 | Absent | Absent | Absent | 2.9 | 1.1 | homogeneous | 1.22 | ≤T2 |
| 479 | male | 60 | 4.34 | 9.14 | descending | 4 | Present | Present | Absent | 3.5 | 1.1 | homogeneous | 0.82 | T3 |
| 480 | male | 70 | 2.75 | 5.53 | rectum | 4 | Present | Absent | Absent | 2.9 | 0.5 | homogeneous | 0.72 | T3 |
| 481 | male | 75 | 4 | 10 | sigmoid | 4 | Absent | Present | Absent | 4.7 | 0.5 | homogeneous | 1.07 | ≤T2 |
| 482 | female | 60 | 10.64 | 6.83 | sigmoid | 4 | Present | Absent | Absent | 4.1 | 0.8 | homogeneous | 0.9 | T3 |
| 483 | female | 66 | 1.43 | 20.97 | rectum | 2 | Present | Absent | Absent | 2.5 | 0.6 | homogeneous | 0.25 | ≤T2 |
| 484 | male | 47 | 1.88 | 8.46 | descending | 4 | Present | Present | Absent | 4.1 | 0.7 | homogeneous | 0.3 | T3 |
| 485 | female | 48 | 2.55 | 7.34 | sigmoid | 4 | Present | Absent | Absent | 2.6 | 0.7 | homogeneous | 0.13 | T3 |
| 486 | female | 47 | 0.46 | 6.42 | rectum | 2 | Absent | Absent | Absent | 1.2 | 0.5 | homogeneous | 2.24 | ≤T2 |
| 487 | female | 62 | 1.41 | 16.26 | rectum | 4 | Present | Present | Absent | 5.1 | 0.7 | homogeneous | 0.94 | T3 |
| 488 | male | 47 | 143.8 | 73.75 | rectum | 4 | Present | Present | Absent | 3.7 | 1.2 | homogeneous | 1.16 | T3 |
| 489 | female | 57 | 0.82 | 6.68 | cecum | 4 | Present | Present | Absent | 4.8 | 1 | homogeneous | 0.8 | ≤T2 |
| 490 | male | 44 | 3.07 | 7.14 | sigmoid | 2 | Present | Absent | Absent | 2.6 | 0.3 | homogeneous | 0.62 | ≤T2 |
| 491 | female | 63 | 11.5 | 10.67 | rectum | 3 | Present | Present | Absent | 4.4 | 1 | homogeneous | 0.85 | T3 |
| 492 | male | 66 | 11.77 | 18.93 | right transverse | 4 | Present | Present | Absent | 6.3 | 0.8 | inhomogeneous | 0.63 | T3 |
| 493 | female | 58 | 1.61 | 17.37 | rectum | 2 | Present | Absent | Absent | 2.8 | 0.4 | inhomogeneous | 1.39 | ≤T2 |
| 494 | male | 59 | 9.47 | 0.6 | ascending | 4 | Absent | Absent | Absent | 4.2 | 1.5 | homogeneous | 0.66 | T3 |
| 495 | female | 48 | 1.91 | 41.58 | right transverse | 4 | Present | Present | Absent | 5.1 | 0.9 | homogeneous | 0.83 | T3 |
| 496 | male | 45 | 2.98 | 22.1 | rectum | 2 | Present | Absent | Absent | 4.3 | 0.7 | homogeneous | 0.42 | T3 |
| 497 | male | 56 | 25.99 | 19.84 | descending | 4 | Present | Present | Absent | 6.5 | 0.9 | homogeneous | 0.44 | T4 |
| 498 | male | 65 | 2 | 19.07 | descending | 2 | Present | Absent | Absent | 4 | 0.6 | homogeneous | 0.84 | ≤T2 |
| 499 | male | 75 | 60.38 | 28.05 | sigmoid | 4 | Present | Present | Absent | 5.1 | 1.2 | inhomogeneous | 0.61 | T3 |
| 500 | male | 48 | 3.89 | 353.7 | left transverse | 4 | Present | Present | Absent | 6.6 | 1.1 | homogeneous | 0.99 | T4 |
| 501 | male | 58 | 8.97 | 5.53 | sigmoid | 4 | Present | Present | Absent | 6.9 | 1.7 | homogeneous | 0.74 | T3 |
| 502 | male | 59 | 1.04 | 9.52 | ascending | 4 | Present | Present | Absent | 4.4 | 1 | inhomogeneous | 1.42 | T3 |
| 503 | male | 81 | 35.01 | 15.03 | cecum | 4 | Absent | Present | Absent | 3.6 | 1 | homogeneous | 1.55 | T4 |
| 504 | female | 73 | 2.78 | 21.59 | rectum | 2 | Present | Present | Absent | 2.3 | 1.6 | homogeneous | 0.76 | ≤T2 |
| 505 | male | 62 | 3.08 | 8.87 | rectum | 4 | Absent | Present | Absent | 3.3 | 0.8 | homogeneous | 0.98 | ≤T2 |
| 506 | female | 67 | 1.06 | 2.03 | rectum | 2 | Absent | Absent | Absent | 1.4 | 0.8 | homogeneous | 1.22 | ≤T2 |
| 507 | male | 67 | 5.88 | 12.16 | sigmoid | 4 | Present | Present | Absent | 4.4 | 0.9 | homogeneous | 0.31 | T3 |
| 508 | male | 66 | 13.46 | 4.36 | sigmoid | 4 | Present | Present | Absent | 8.1 | 1.6 | homogeneous | 0.3 | T3 |
| 509 | female | 53 | 0.77 | 20.53 | sigmoid | 3 | Present | Absent | Absent | 2.6 | 0.6 | homogeneous | 2.6 | T3 |
| 510 | male | 65 | 3.21 | 23.35 | sigmoid | 4 | Present | Absent | Absent | 5 | 1 | homogeneous | 0.99 | T3 |
| 511 | female | 47 | 3.24 | 33.38 | sigmoid | 3 | Present | Present | Absent | 4.1 | 1 | homogeneous | 0.39 | T3 |
| 512 | female | 53 | 8.22 | 45.63 | sigmoid | 4 | Present | Present | Absent | 4.2 | 1.2 | inhomogeneous | 1.46 | T3 |
| 513 | female | 64 | 13.59 | 10.45 | ascending | 4 | Present | Present | Absent | 3.8 | 0.8 | homogeneous | 0.88 | T3 |
| 514 | female | 58 | 4.12 | 118.1 | right transverse | 4 | Present | Present | Absent | 7 | 1.6 | inhomogeneous | 0.48 | T4 |
| 515 | female | 70 | 3.13 | 5.96 | ascending | 4 | Present | Present | Absent | 5 | 1.2 | homogeneous | 0.94 | T4 |
| 516 | male | 70 | 41.25 | 0.6 | rectum | 4 | Present | Present | Present | 4.6 | 1.5 | homogeneous | 0.36 | T4 |
| 517 | male | 49 | 1.73 | 4.82 | rectum | 4 | Present | Present | Absent | 5.6 | 0.8 | homogeneous | 1.26 | T4 |
| 518 | female | 49 | 11.38 | 89.53 | rectum | 4 | Present | Present | Absent | 6.8 | 0.6 | inhomogeneous | 0.82 | T4 |
| 519 | male | 50 | 17.87 | 14.92 | right transverse | 4 | Present | Present | Absent | 4.7 | 1.1 | homogeneous | 0.83 | T4 |
| 520 | female | 30 | 2.72 | 182.61 | right transverse | 4 | Present | Present | Absent | 5.6 | 1.4 | homogeneous | 1.71 | T4 |
| 521 | female | 61 | 2.89 | 1000 | cecum | 4 | Present | Present | Present | 1.3 | 2.8 | inhomogeneous | 0.4 | T4 |
| 522 | female | 57 | 1.53 | 6.72 | rectum | 3 | Present | Absent | Absent | 2.8 | 0.5 | homogeneous | 1.16 | T4 |
| 523 | male | 53 | 4.33 | 15.68 | rectum | 4 | Present | Present | Absent | 6 | 1.1 | inhomogeneous | 0.77 | T4 |
| 524 | female | 53 | 16.4 | 111 | rectum | 4 | Present | Absent | Absent | 4.4 | 0.7 | inhomogeneous | 1.5 | T4 |
| 525 | male | 65 | 2.81 | 3.73 | rectum | 4 | Present | Present | Absent | 6.6 | 1.1 | inhomogeneous | 0.93 | T4 |
| 526 | female | 61 | 1.71 | 5.54 | rectum | 4 | Present | Present | Present | 5.3 | 1 | homogeneous | 0.92 | T4 |
| 527 | male | 59 | 4.8 | 11.62 | rectum | 3 | Present | Absent | Absent | 4 | 2.2 | homogeneous | 1.36 | T4 |
| 528 | female | 33 | 1.41 | 13.28 | rectum | 4 | Present | Present | Absent | 4.8 | 1.3 | homogeneous | 0.77 | T4 |
| 529 | male | 53 | 35.39 | 6.63 | rectum | 4 | Present | Absent | Absent | 4.3 | 1 | homogeneous | 0.95 | T4 |
| 530 | female | 38 | 1.59 | 5.6 | rectum | 4 | Absent | Absent | Absent | 4 | 0.7 | homogeneous | 0.95 | T4 |
| 531 | male | 60 | 14.25 | 0.6 | rectum | 4 | Absent | Absent | Absent | 4 | 0.9 | homogeneous | 0.84 | T4 |
| 532 | male | 54 | 4.85 | 8.15 | rectum | 3 | Present | Absent | Absent | 3 | 1.3 | homogeneous | 0.51 | ≤T2 |
| 533 | male | 67 | 5.62 | 5.66 | rectum | 2 | Present | Absent | Absent | 3.7 | 0.8 | homogeneous | 0.94 | ≤T2 |
| 534 | male | 67 | 2.57 | 12.83 | rectum | 2 | Present | Absent | Absent | 1.7 | 0.7 | homogeneous | 0.61 | ≤T2 |
| 535 | female | 51 | 1.01 | 7.22 | sigmoid | 4 | Present | Absent | Absent | 4.6 | 1.2 | homogeneous | 0.47 | ≤T2 |
| 536 | male | 61 | 1.52 | 7.73 | rectum | 2 | Absent | Absent | Absent | 3.5 | 0.7 | homogeneous | 0.25 | ≤T2 |
| 537 | male | 59 | 1.64 | 14.82 | right transverse | 1 | Absent | Absent | Absent | 0.9 | 0.5 | homogeneous | 1.82 | ≤T2 |
| 538 | female | 43 | 0.48 | 15.2 | rectum | 3 | Absent | Absent | Absent | 4.2 | 1.8 | homogeneous | 0.56 | ≤T2 |
| 539 | male | 59 | 0.53 | 6.08 | rectum | 2 | Absent | Absent | Absent | 1.7 | 0.9 | homogeneous | 0.55 | ≤T2 |
| 540 | male | 43 | 12.01 | 3.46 | rectum | 4 | Present | Absent | Absent | 4.7 | 2.3 | homogeneous | 0.53 | ≤T2 |
| 541 | female | 53 | 0.75 | 3.68 | rectum | 2 | Absent | Absent | Absent | 3.6 | 0.9 | homogeneous | 0.76 | ≤T2 |
| 542 | male | 53 | 2.64 | 6.19 | sigmoid | 4 | Present | Absent | Absent | 5.5 | 2.6 | inhomogeneous | 0.85 | ≤T2 |
| 543 | male | 60 | 3.88 | 12.31 | rectum | 1 | Absent | Absent | Absent | 0.8 | 0.5 | homogeneous | 0.85 | ≤T2 |
| 544 | male | 67 | 2.2 | 15.6 | rectum | 2 | Present | Absent | Absent | 1.6 | 0.7 | homogeneous | 1.08 | ≤T2 |
| 545 | male | 70 | 4.36 | 6.95 | rectum | 4 | Present | Absent | Absent | 4.1 | 3.2 | inhomogeneous | 0.86 | ≤T2 |
| 546 | male | 59 | 2.75 | 12.44 | rectum | 2 | Absent | Absent | Absent | 2.3 | 0.7 | homogeneous | 0.79 | ≤T2 |
| 547 | male | 79 | 2.7 | 39.06 | sigmoid | 2 | Present | Absent | Absent | 3.8 | 1 | inhomogeneous | 1.05 | ≤T2 |
| 548 | female | 63 | 3.55 | 4.79 | rectum | 2 | Absent | Absent | Absent | 1.9 | 1 | homogeneous | 0.85 | ≤T2 |
| 549 | female | 64 | 1.43 | 5.31 | rectum | 3 | Absent | Absent | Absent | 2.1 | 0.5 | homogeneous | 4.21 | ≤T2 |
| 550 | male | 68 | 4.81 | 3.92 | rectum | 2 | Absent | Absent | Absent | 1 | 0.7 | homogeneous | 1.36 | ≤T2 |
| 551 | female | 61 | 0.88 | 16.21 | rectum | 2 | Absent | Absent | Absent | 1.9 | 0.5 | homogeneous | 0.89 | ≤T2 |
| 552 | male | 59 | 2.93 | 13.14 | rectum | 2 | Present | Absent | Absent | 2.6 | 0.6 | homogeneous | 0.93 | ≤T2 |
| 553 | female | 55 | 6.87 | 45.08 | rectum | 3 | Absent | Absent | Absent | 3.2 | 0.7 | homogeneous | 1.1 | ≤T2 |
| 554 | female | 81 | 4.47 | 51.59 | cecum | 2 | Absent | Absent | Absent | 2.4 | 0.8 | homogeneous | 0.65 | ≤T2 |
| 555 | female | 66 | 1.27 | 11.58 | sigmoid | 3 | Present | Absent | Absent | 3.3 | 1.1 | homogeneous | 0.72 | ≤T2 |
| 556 | male | 83 | 1.8 | 7.73 | sigmoid | 3 | Present | Absent | Absent | 3.4 | 1 | homogeneous | 0.63 | ≤T2 |
| 557 | male | 71 | 1.56 | 5.99 | sigmoid | 4 | Absent | Absent | Absent | 2.8 | 0.5 | homogeneous | 0.53 | ≤T2 |
| 558 | female | 60 | 4.96 | 18.51 | rectum | 2 | Absent | Absent | Absent | 2.4 | 0.7 | homogeneous | 1.38 | ≤T2 |
| 559 | female | 54 | 0.2 | 67.91 | rectum | 3 | Absent | Absent | Absent | 1.7 | 1.1 | homogeneous | 0.69 | ≤T2 |
| 560 | female | 45 | 3.89 | 15.15 | rectum | 4 | Absent | Absent | Absent | 3.8 | 1.6 | homogeneous | 1.01 | ≤T2 |
| 561 | female | 65 | 1.01 | 5.84 | rectum | 3 | Absent | Absent | Absent | 2.2 | 1.5 | homogeneous | 0.93 | ≤T2 |
| 562 | male | 71 | 3.03 | 10.59 | sigmoid | 3 | Absent | Absent | Absent | 5.2 | 3.8 | homogeneous | 0.53 | ≤T2 |
| 563 | female | 62 | 11.7 | 17.18 | rectum | 3 | Present | Absent | Absent | 1.8 | 0.9 | homogeneous | 1.51 | ≤T2 |
| 564 | female | 62 | 4.01 | 7.11 | rectum | 3 | Present | Absent | Absent | 3.1 | 0.6 | homogeneous | 0.99 | ≤T2 |
| 565 | male | 79 | 2.58 | 21.01 | rectum | 3 | Absent | Absent | Absent | 2.6 | 0.8 | homogeneous | 0.92 | ≤T2 |
| 566 | male | 59 | 13.56 | 14.24 | rectum | 4 | Absent | Absent | Absent | 6.5 | 1.3 | homogeneous | 0.73 | ≤T2 |
| 567 | male | 68 | 3.37 | 9.6 | rectum | 3 | Absent | Absent | Absent | 4.1 | 3.6 | homogeneous | 0.77 | ≤T2 |
| 568 | male | 91 | 2.69 | 15.55 | descending | 3 | Present | Absent | Absent | 5.8 | 11.4 | homogeneous | 1.24 | ≤T2 |
| 569 | male | 58 | 5.04 | 52.36 | rectum | 2 | Present | Present | Absent | 2.8 | 0.5 | homogeneous | 1.29 | ≤T2 |
| 570 | male | 68 | 1.08 | 10.5 | sigmoid | 3 | Absent | Absent | Absent | 3.7 | 1.3 | homogeneous | 0.45 | ≤T2 |
| 571 | male | 66 | 2.63 | 5.43 | rectum | 3 | Present | Absent | Absent | 2.5 | 1.6 | homogeneous | 0.72 | ≤T2 |
| 572 | female | 66 | 117.6 | 85.74 | sigmoid | 4 | Present | Present | Absent | 5.1 | 1.2 | homogeneous | 1.56 | T4 |
| 573 | male | 66 | 453.2 | 1000 | ascending | 4 | Present | Present | Absent | 8 | 1 | inhomogeneous | 0.41 | T4 |
| 574 | female | 56 | 30 | 80 | ascending | 4 | Present | Present | Absent | 6.8 | 2.3 | inhomogeneous | 0.36 | T4 |
| 575 | male | 82 | 6.99 | 25.9 | cecum | 4 | Present | Present | Present | 4.5 | 1.8 | inhomogeneous | 0.86 | T4 |
| 576 | female | 63 | 10 | 30 | ascending | 4 | Present | Present | Absent | 3.4 | 1.2 | inhomogeneous | 1.06 | T4 |
| 577 | female | 69 | 21.14 | 8.28 | cecum | 4 | Present | Present | Absent | 4.5 | 0.8 | inhomogeneous | 0.74 | T4 |
| 578 | female | 55 | 101 | 419.7 | sigmoid | 4 | Present | Present | Absent | 9.5 | 1.1 | inhomogeneous | 0.75 | T4 |
| 579 | female | 60 | 13.3 | 61.45 | ascending | 4 | Present | Present | Absent | 8.8 | 2.6 | inhomogeneous | 1.16 | T4 |
| 580 | female | 62 | 152.8 | 8.49 | rectum | 4 | Present | Present | Present | 9 | 1.6 | inhomogeneous | 0.86 | T4 |
| 581 | female | 39 | 1.02 | 20.42 | ascending | 4 | Present | Present | Absent | 9.4 | 1.9 | inhomogeneous | 1.41 | T4 |
| 582 | male | 66 | 1.27 | 10.54 | sigmoid | 4 | Present | Present | Absent | 8.3 | 2.4 | homogeneous | 0.51 | T4 |
| 583 | female | 52 | 20.98 | 32.95 | sigmoid | 4 | Present | Present | Absent | 4.7 | 1.8 | inhomogeneous | 1.39 | T4 |
| 584 | male | 84 | 6.7 | 26.16 | sigmoid | 4 | Present | Present | Present | 8.6 | 1.6 | homogeneous | 0.79 | T4 |
| 585 | female | 58 | 15.44 | 17.92 | ascending | 4 | Present | Present | Absent | 4.3 | 1.1 | homogeneous | 0.88 | T4 |
| 586 | male | 43 | 63.49 | 6.04 | descending | 4 | Present | Present | Absent | 5.3 | 0.9 | homogeneous | 0.35 | T4 |
| 587 | male | 73 | 11.69 | 14.47 | rectum | 4 | Present | Present | Absent | 4.6 | 1.3 | homogeneous | 0.55 | T4 |
| 588 | male | 43 | 20.25 | 790.4 | sigmoid | 4 | Present | Present | Absent | 4.5 | 1.1 | homogeneous | 0.69 | T4 |
| 589 | male | 51 | 0.79 | 13.87 | cecum | 4 | Present | Present | Present | 8.4 | 2.2 | inhomogeneous | 0.58 | T4 |
| 590 | male | 68 | 4.17 | 4.32 | cecum | 4 | Present | Present | Absent | 7.5 | 1.8 | inhomogeneous | 0.71 | T4 |
| 591 | male | 68 | 2.36 | 10.19 | left transverse | 4 | Present | Present | Absent | 7.6 | 1.3 | inhomogeneous | 0.23 | T4 |
| 592 | male | 78 | 5.55 | 222.7 | descending | 4 | Present | Present | Absent | 3.4 | 0.7 | homogeneous | 0.67 | T4 |
| 593 | male | 57 | 11.46 | 261.3 | cecum | 4 | Present | Present | Absent | 6 | 3.1 | inhomogeneous | 0.67 | T4 |
| 594 | female | 61 | 13.75 | 90.36 | right transverse | 4 | Present | Present | Present | 7.5 | 1.1 | homogeneous | 1.27 | T4 |
| 595 | female | 52 | 4.73 | 10.61 | sigmoid | 4 | Present | Present | Absent | 6 | 1.2 | inhomogeneous | 1.21 | T4 |
| 596 | male | 38 | 19.36 | 6.93 | right transverse | 4 | Present | Present | Absent | 7.2 | 1.6 | inhomogeneous | 0.81 | T4 |
| 597 | female | 74 | 41.48 | 123.6 | descending | 4 | Present | Present | Absent | 8.2 | 0.8 | inhomogeneous | 0.99 | T4 |
| 598 | male | 52 | 1.41 | 289.9 | right transverse | 4 | Present | Present | Absent | 3.6 | 1.2 | homogeneous | 0.95 | T4 |
| 599 | male | 64 | 232.52 | 361.61 | descending | 4 | Present | Present | Absent | 5.3 | 1.1 | inhomogeneous | 0.76 | T4 |
| 600 | female | 80 | 58.87 | 800 | right transverse | 4 | Present | Present | Absent | 6.1 | 0.9 | inhomogeneous | 0.9 | T4 |
| 601 | female | 49 | 16.12 | 127.9 | cecum | 4 | Present | Present | Absent | 3.3 | 1.4 | homogeneous | 0.74 | T4 |
| 602 | female | 48 | 59.95 | 291.41 | left transverse | 4 | Present | Present | Absent | 5 | 1.2 | homogeneous | 0.58 | T4 |
| 603 | female | 52 | 2.06 | 12.08 | cecum | 4 | Present | Present | Present | 5.5 | 2.1 | inhomogeneous | 1.3 | T4 |
| 604 | male | 59 | 6.31 | 20.05 | rectum | 4 | Present | Absent | Absent | 3.1 | 0.9 | homogeneous | 0.95 | T4 |
| 605 | female | 69 | 1000 | 31.25 | sigmoid | 4 | Present | Present | Absent | 4.2 | 1.4 | inhomogeneous | 0.46 | T4 |
| 606 | male | 46 | 2.47 | 19.63 | ascending | 4 | Present | Present | Absent | 2.7 | 1.4 | homogeneous | 0.9 | T4 |
| 607 | female | 67 | 2.24 | 21.18 | sigmoid | 4 | Present | Absent | Absent | 2.7 | 1 | homogeneous | 0.82 | T4 |
| 608 | male | 58 | 1.63 | 5.7 | rectum | 4 | Present | Present | Absent | 5 | 3 | homogeneous | 0.77 | T4 |
| 609 | female | 58 | 3.73 | 22.69 | ascending | 4 | Present | Present | Present | 9.9 | 2 | inhomogeneous | 0.53 | T4 |
| 610 | female | 58 | 1.23 | 12.96 | ascending | 4 | Present | Present | Absent | 4.4 | 1.7 | inhomogeneous | 0.55 | T4 |
| 611 | female | 70 | 3.13 | 5.96 | ascending | 4 | Present | Present | Absent | 4.3 | 1.1 | homogeneous | 1.01 | T4 |
|  |  |  |  |  |  |  |  |  |  |  |  |  |  |  |
